# Genetic variants associated with physiological and biochemical indicators: A multi‐centre whole‐exome sequencing study of Chinese healthy participants

**DOI:** 10.1002/ctm2.70300

**Published:** 2025-04-09

**Authors:** Zhe Wang, Zhiyan Liu, Guangyan Mu, Qiufen Xie, Shuang Zhou, Zining Wang, Yimin Cui, Qian Xiang, IMPACT Study Group

**Affiliations:** ^1^ Institute of Clinical Pharmacology Peking University First Hospital Beijing China; ^2^ Department of Pharmacy Peking University First Hospital Beijing China

Dear Editor,

Disease onset and progression manifest through quantifiable alterations in organ function biomarkers, including hepatic, renal and lipid profiles. Even within normal reference ranges, these indicators could predict disease risk.^1^ Physiological and biochemical profiles in Asians display distinct characteristics compared to those in Caucasians.^2^ Identifying factors that influence these markers in Asian populations may therefore enhance early disease prediction and intervention, improving clinical outcomes.

Given the heritability of physiological and biochemical indicators,^3^ linking genetic variations with these indicators differences through genome‐wide association studies (GWAS) has become a highly promising approach, particularly in lipid metabolism.^4^ However, databases such as UK Biobank and FinnGen present limitation due to ethnic diversity and lack of stringent screening. This study employed whole‐exome sequencing to analyse genes associated with key physiological indicators in clinically screened Chinese healthy participants, providing insights into their genetic correlations.

A total of 778 participants in IMPACT study were included in this cohort.^5^, ^6^, ^7^, ^8^ Briefly, healthy volunteers were enrolled in this study based on the absence of clinically significant abnormalities in blood pressure, heart rate, routine blood, blood biochemistry, chest X‐ray, urinalysis and electrocardiogram, as well as confirmation of no infectious diseases or non‐pregnancy status. Figure 1 shows the flow diagram. The physiological and biochemical indicators of participants are presented in Table 1. In discovery cohort, correlation analysis and linear regression were conducted to choose the covariates (Figure S1 and Table S1). Consequently, age, male proportion and body mass index were included as covariates in subsequent genetic association analyses. Identity‐by‐descent analysis excluded first‐degree relatives (Proportion of identity by descent: PI_HAT ≤ .5) prior to genetic modeling.

**FIGURE 1:**
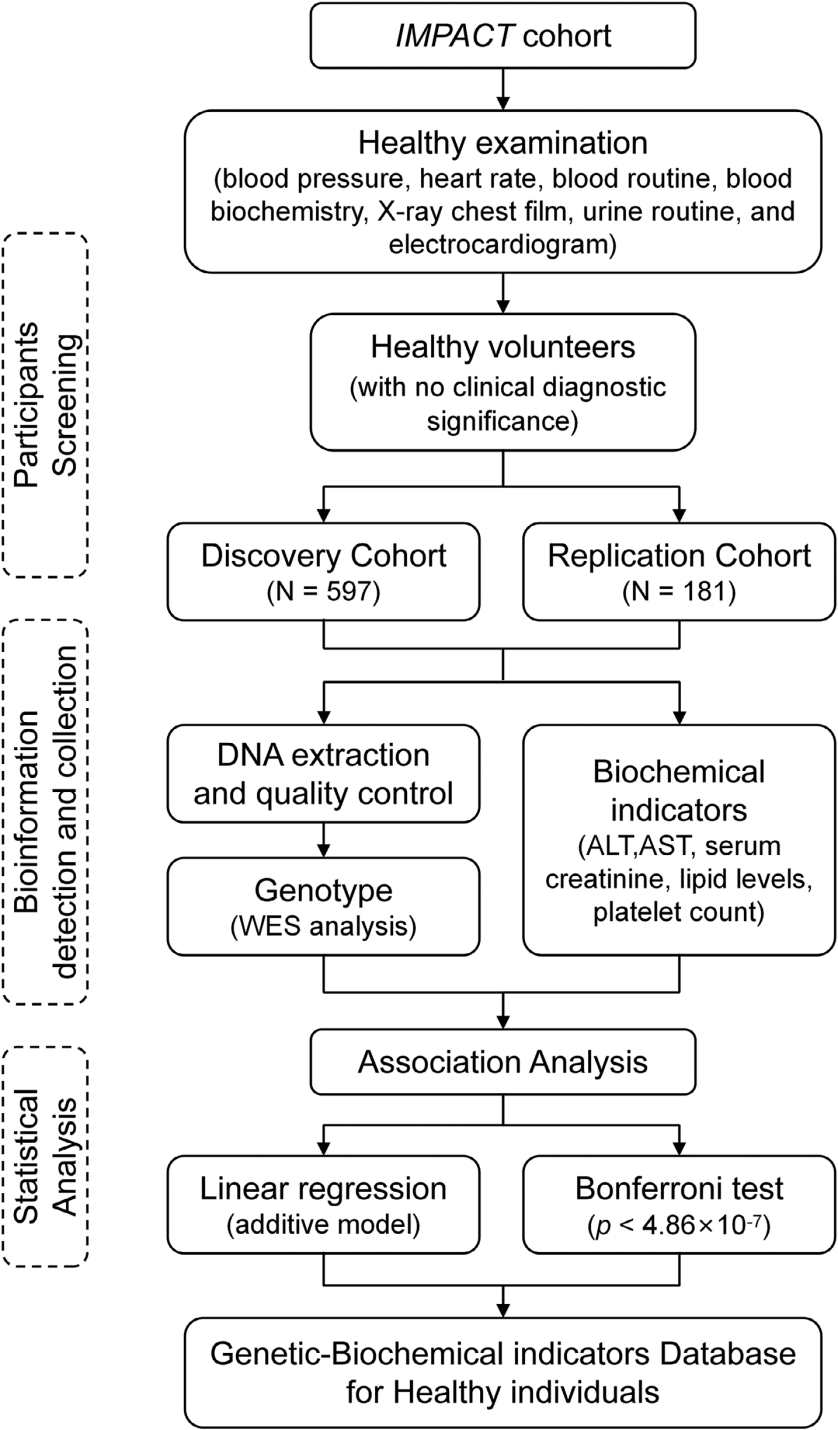
Flow chart summarised the steps and results in this study. This study enrolled healthy subjects without clinically diagnosed significance after health examination in the IMPACT study. A total of 778 healthy participants were included, with 597 served as the discovery cohort and 181 served as the replication cohort. Whole‐exome sequencing was used to detect genotype. Key clinical indicators—alanine aminotransferase (ALT), aspartate aminotransferase (AST), serum creatinine, lipid levels (low‐density lipoprotein, high‐density lipoprotein, total cholesterol and triglycerides) and platelet counts were collected. Genetic‒biochemical association analysis was performed by linear regression, assuming an additive genetic model. According to Bonferroni test, the significance level for successful replication was set at *p* = .05/102 848 = 4.86 × 10^−7^ of single nucleotide polymorphisms (SNPs) for which replication was attempted. Considering sample size, SNPs with *p*‐value <10^−4^ were also presented.

**TABLE 1:** Characteristic of enrolled healthy participants.

Characteristic	Discovery cohort (*N* = 597), mean ± standard deviation	Replication cohort (*N* = 181), mean ± standard deviation	*p*‐Value
Age	28.71 ± 8.26	29.82 ± 6.65	.064
18 ≤ age < 30	61.24% (371/597)	53.04% (96/181)	
30 ≤ age < 40	24.29% (145/597)	35.91% (65/181)	
40 ≤ age < 50	12.2% (73/597)	11.05% (20/181)	
50 ≤ age < 60	1.34% (8/597)	.00% (0/181)	
Male	67.67% (193/597)	64.64% (64/181)	.448
Weight (kg)	62.65 ± 7.63	62.23 ± 7.35	.540
Body mass index	22.65 ± 1.94	22.40 ± 1.88	.113
Alanine aminotransferase (U/L)	16.83 ± 9.52	16.65 ± 8.91	.821
Aspartate aminotransferase (U/L)	18.47 ± 4.91	19.34 ± 4.58	.034
Serum creatinine (µmol/L)	74.47 ± 16.46	66.80 ± 12.85	<.001
Platelet (10^9^/L)	254.07 ± 55.65	Not record	No data
Total cholesterol (mmol/L)	4.32 ± .73	4.10 ± .70	.002
Triglyceride (mmol/L)	1.06 ± .51	1.13 ± .52	.183
Low‐density lipoprotein (mmol/L)	2.47 ± .62	2.41 ± .51	.522
High‐density lipoprotein (mmol/L)	1.37 ± .28	1.30 ± .33	.144

To investigate genetic variants associated with liver function, genetic association analysis was conducted in total cholesterol (TC), triglyceride (TG), low‐density lipoprotein (LDL), high‐density lipoprotein (HDL), alanine aminotransferase (ALT) and aspartate aminotransferase (AST). The Manhattan plot of discovery cohort is shown in Figure 2. Single nucleotide polymorphisms (SNPs) correlated with liver function and disease are listed in Tables S2‐S7.  Notably, *RBM19* rs117916372 was associated with both ALT and AST. Compared with AA carriers of rs117916372, homozygous GG carriers exhibited lower level of ALT (AA vs. GG: 16.20 ± 8.66 U/L vs. 12.75 ± 4.43 U/L, *p *= 1.53 × 10^−7^) and AST (AA vs. GG: 18.38 ± 4.61 U/L vs. 15.00 ± 4.24 U/L, *p = *2.56 × 10^−10^). Rs3851 on *TM4SF5* exhibited a correlation with ALT and AST levels in both the discovery and replication cohorts (Table S8 ).

**FIGURE 2:**
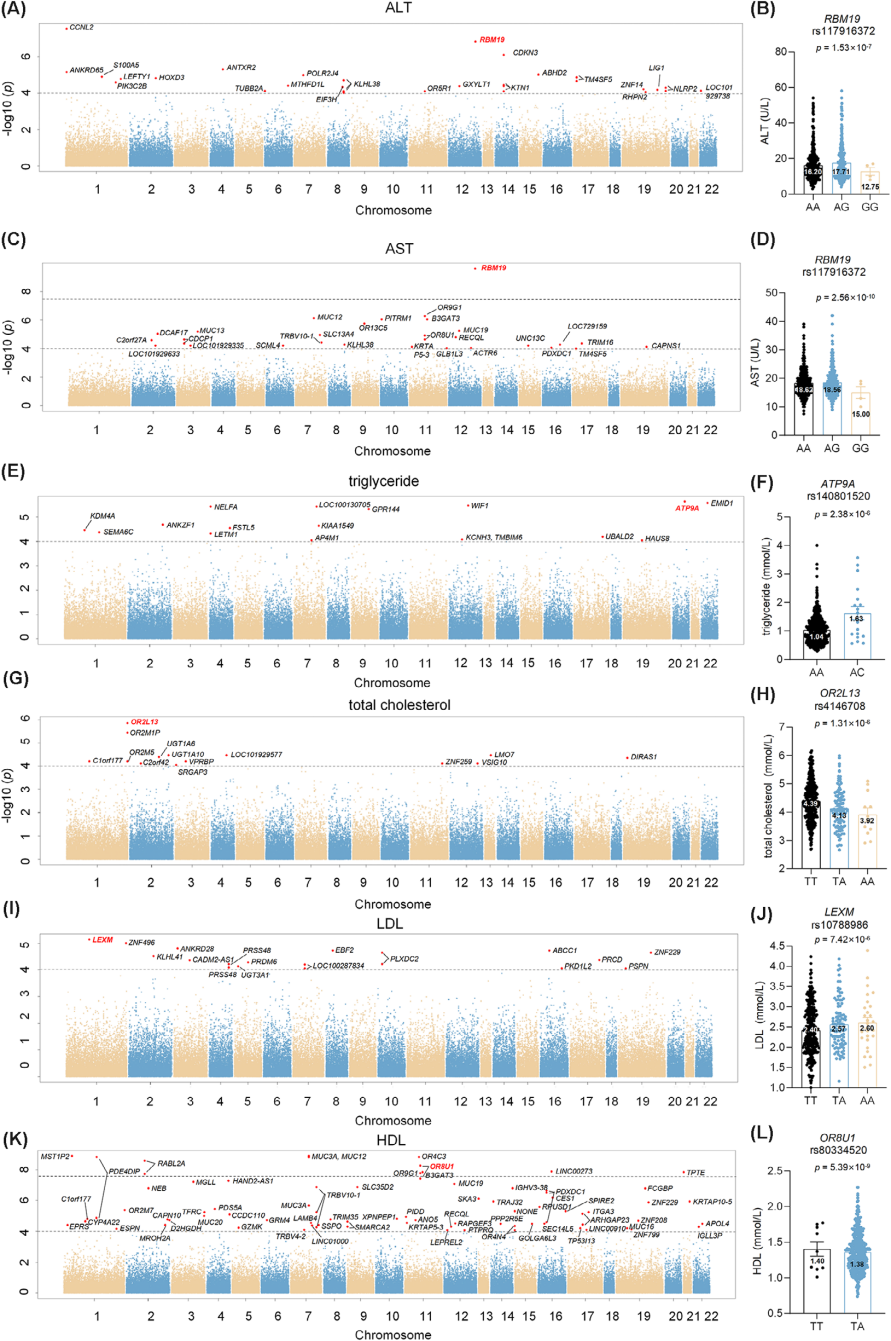
Genetic variants correlated with liver function. (A) Manhattan plot of the genetic association analysis with alanine aminotransferase (ALT). (B) ALT level in carriers with rs117916372 on *RBM19* gene. (C) Manhattan plot of the genetic association analysis with aspartate aminotransferase (AST). (D) AST level in carriers with rs117916372 on *RBM19* gene. (E) Manhattan plot of the genetic association analysis with triglyceride. (F) Triglyceride level in carriers with rs140801520 on *ATP9A* gene. (G) Manhattan plot of the genetic association analysis with total cholesterol. (H) Total cholesterol level in carriers with rs4146708 on *OR2L13* gene. (I) Manhattan plot of the genetic association analysis with low‐density lipoprotein (LDL). (J) LDL level in carriers with rs10788986 on *LEXM* gene. (K) Manhattan plot of the genetic association analysis with high‐density lipoprotein (HDL). (L) HDL level in carriers with rs80334520 on *OR8U1* gene.

In the genome‐wide analyses of TG, *ATP9A* rs140801520 was most significantly associated with TG levels (*p = *2.38 × 10^−6^). *UBALD2* rs712833 exhibited a correlation with TG levels in both the discovery and replication cohorts (Table S8). For TC, *OR2L13* rs4146708 was primarily correlated with cholesterol variation (*p = *1.31 × 10^−6^). For LDL, *LEXM* rs10788986 showed a notable association with LDL (*p = *7.42 × 10^−6^). Regarding HDL level, multiple SNPs on *OR8U1* were associated (*p = *5.39 × 10^−9^). Notably, *OR8U1* also has a potential correlation with AST level (*p = *1.32 × 10^−5^). Compared with TT carriers of rs80334520, heterozygous TA carriers exhibited lower level of AST (TT vs. TA: 23.01 ± 9.35 U/L vs. 18.43 ± 4.63 U/L) and HDL (TT vs. TA: 1.41 ± .28 mmol/L vs. 1.38 ± .29 mmol/L). Elevated serum AST and HDL levels suggests hepatocyte damage or chronic inflammation.^9^ The association of *OR8U1* with both AST and HDL levels indicted the potential role of olfactory receptor OR8U1 in liver metabolic‐inflammatory regulation, requiring further in vivo and in vitro validation.

Genetic association analysis of serum creatinine is shown in Figure 3. SNPs correlated with serum creatinine are listed in Table S9. Among them, multiple SNPs located on *NBPF1*, *MST1P2*, *NBPF10*, *SDHAP2*, *FRG1*, *FRG1B*, *MUC3A* and *MUC6*. A mutation in rs3901679 on *NBPF1* from the T to C allele was associated with a significant increase in serum creatinine levels (TT vs. TC: 72.96 ± 16.31 µmol/L vs. 86.56 ± 12.21 µmol/L). In addition, rs80068592, rs140898464 and rs61734664 exhibited a potential correlation with creatinine in both the discovery and replication cohorts, as shown in Table S8.

**FIGURE 3:**
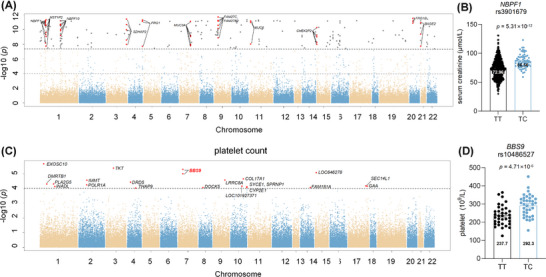
Genetic variants correlated with serum creatinine and platelet count. (A) Manhattan plot of the genetic association analysis with serum creatinine. (B) Creatinine level in carriers with rs3901679 on *NBPF1* gene. (C) Manhattan plot of the genetic association analysis with platelet count. (D) Platelet count in carriers with rs10486527 on *BBS9* gene.

To investigate genetic variants associated with hematopoiesis, genetic association analysis was conducted with platelet count (Table S10). Several SNPs on *BBS9*, *POLR1A* and *SPRNP1* genes demonstrated significant correlations with platelet count in Figure 3. Specifically, allele change from T to C on rs10486527 was associated with an increased platelet count (TT vs. TC: 244.30 ± 50.36 × 10^9^/L vs. 292.83 ± 60.15 × 10^9^/L, *p *= 4.71 × 10^−6^).

We also conducted cross‐validation of SNPs reported in previous GWAS. No statistical significant associations was observed (Table S11). Potential significant associations were presented as follows: *ZNF646* rs749671 with TG (*p = *.0017), *TM6SF2* rs58542926 with ALT (*p* = .0058), *DHODH* rs2288002 with LDL (*p* = .0026) and *PCNXL3* rs12801636 with TG (*p* = .044). Although prior findings were not fully replicated in this study, correlation analyses revealed concordant effect directions with published data. However, larger effect sizes (*β* and SE) were observed in this cohort, potentially attributable to its comparatively smaller sample size. Meanwhile, the inability of this study to replicate the majority of previously reported SNPs may be attributed to several factors. First, most prior cohorts included both patients and healthy controls, potentially introducing heterogeneity. Second, previous studies were predominantly conducted in European populations. Ethnic distinctions between Caucasian and East Asian groups, such as differences in minor allele frequencies, may account for the observed discrepancies.

This study has certain limitations. Baseline differences were observed between the discovery cohort and the validation cohort, potentially attributed to the limited sample size in the validation cohort. This potentially explain the inability to replicate discovery cohort results. This study did not account for participants' exercise habits or dietary patterns, which may introduce confounding effects on clinical indicators beyond genetic factors. Participants were healthy individuals with a younger average age and normal physiological and biochemical indicators compared to typical clinical patient populations. This study was conducted only among Chinese populations. Cross‐ethnic comparisons study and functional validation are needed to confirm the role of the genes.

## 1 CONCLUSION

This study established associations between genetic variants and biochemical indicators through a database of healthy individuals. Our cohort identified genes correlated with biochemical indicators, offering promising biomarkers and therapeutic insights for future clinical disease prediction, diagnosis and drug development.

## AUTHOR CONTRIBUTIONS

*Conception and design*: Yimin Cui and Qian Xiang. *Provision of participants and study materials*: Zhe Wang, Zhiyan Liu, Qiufen Xie, Guangyan Mu, Shuang Zhou, Zining Wang and IMPACT study group. *Collection of data*: Zhe Wang and Zhiyan Liu. *Data analysis and interpretation*: Zhe Wang and Qian Xiang. *Manuscript writing*: Zhe Wang and Qian Xiang. All the authors have read and approved the final manuscript.

## CONFLICT OF INTEREST STATEMENT

The authors declare they have no conflicts of interest.

## ETHICS STATEMENT

All studies were approved by the independent ethics committee of Peking University First Hospital and all participating centres. Subjects were informed before study and provided written informed consent. This study was registered on ClinicalTrial.org with the registration numbers NCT03161496 and NCT03161002.

## Supporting information



Supporting Information

Supporting Information

## Data Availability

The data that support the findings of this study are deposited in the National Population Health Data Center (https://www.ncmi.cn) and available from the corresponding author upon reasonable request (doi:10.12213/11.A0028.202009.338.V1.0).
