# Supplementary material for: Genetic variants associated with physiological and biochemical indicators: A multi‐centre whole‐exome sequencing study of Chinese healthy participants
Source: Clin Transl Med. 2025 Apr 9;15(4):e70300. doi: 10.1002/ctm2.70300 (PMC11982517; doi:10.1002/ctm2.70300)
Supplement: Supplementary file 1 — Supporting Information [file CTM2-15-e70300-s001.docx]

**Supplementary Materials**

[Figure S1 Correlation of characteristic indicators. 1](#_Toc32333)

[Figure S2 mRNA expression of genetic variation according to eQTL 2](#_Toc16613)

[Figure S3 Q-Q plot of genome-wide association 3](#_Toc14632)

[Table S1 Linear regression independent variable of covariates and indicators 4](#_Toc31146)

[Table S2 Single nucleotide polymorphism correlated to ALT 6](#_Toc16365)

[Table S3 Single nucleotide polymorphism correlated to AST 7](#_Toc2748)

[Table S3 Single nucleotide polymorphism correlated to triglyceride 8](#_Toc29371)

[Table S5 Single nucleotide polymorphism correlated to total cholesterol 10](#_Toc15849)

[Table S6 Single nucleotide polymorphism correlated to low density lipoprotein 11](#_Toc32143)

[Table S7 Single nucleotide polymorphism correlated to high density lipoprotein 12](#_Toc6911)

[Table S8 Genetic Variants detected in both discovery and replication cohorts 16](#_Toc11705)

[Table S9 Single nucleotide polymorphism correlated to serum creatinine 17](#_Toc29663)

[Table S10 Single nucleotide polymorphism correlated to platelet count 26](#_Toc10272)


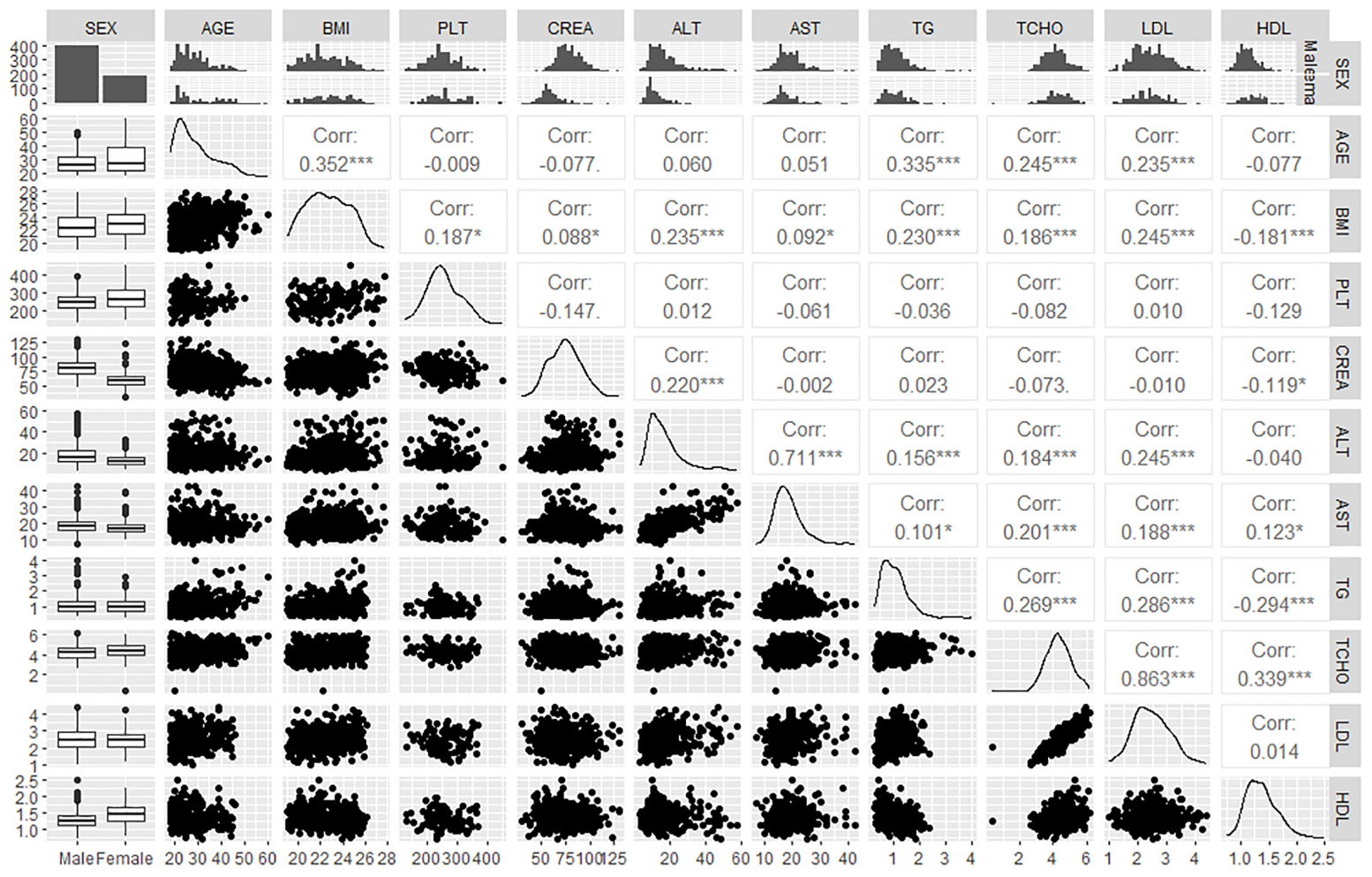


**Figure S1** Correlation of characteristic indicators.

* represent *p* < 0.05, ** represent *p* < 0.01, *** represent *p* < 0.001


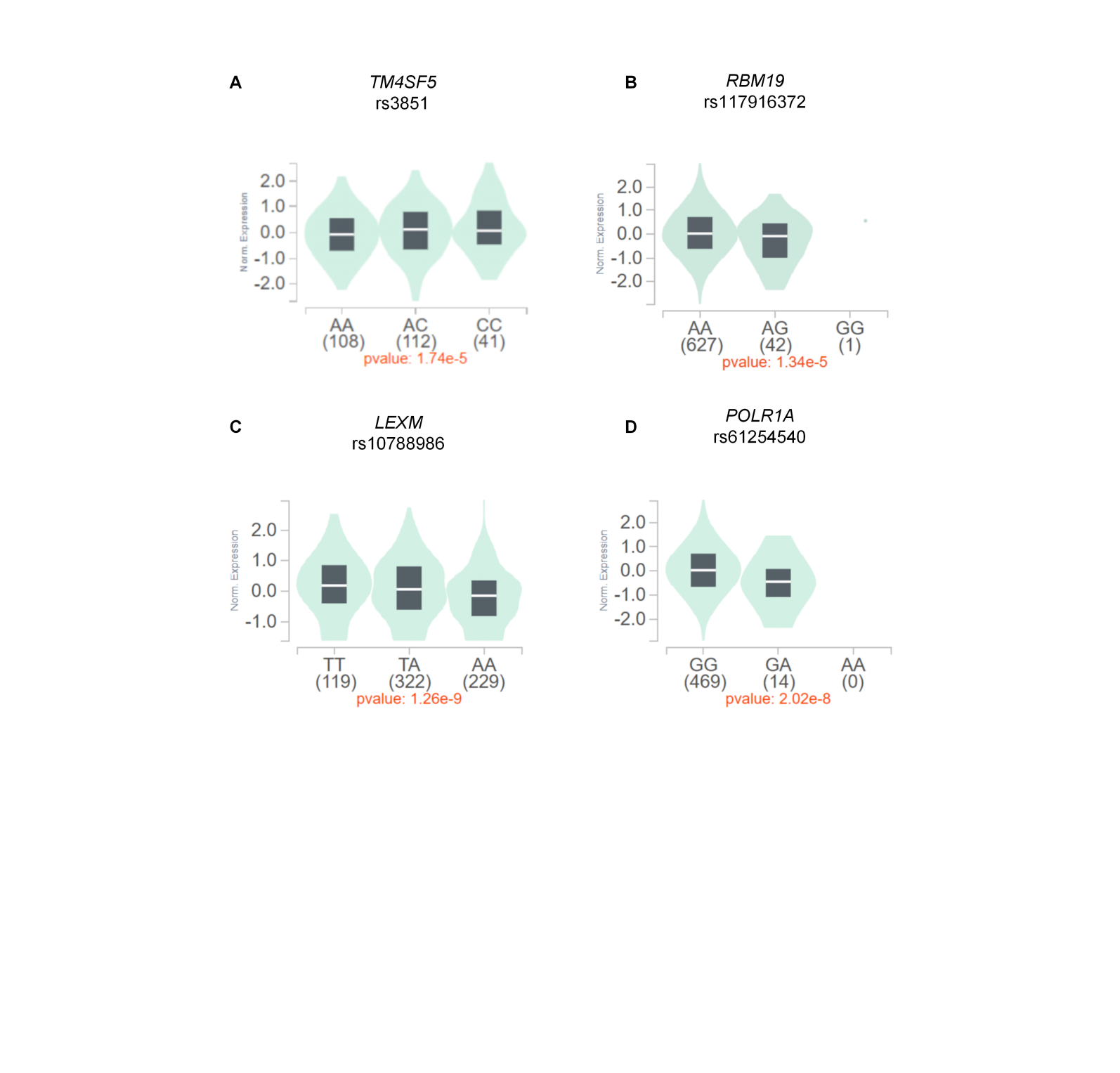


**Figure S2** mRNA expression of genetic variation according to eQTL

1. mRNA level of TM4SF5 with different genotype of rs3851. (B) mRNA level of RBM19 with different genotype of rs117916372. (C) mRNA level of LEXM with different genotype of rs10788986. (D) mRNA level of POLR1A with different genotype of rs61254540.


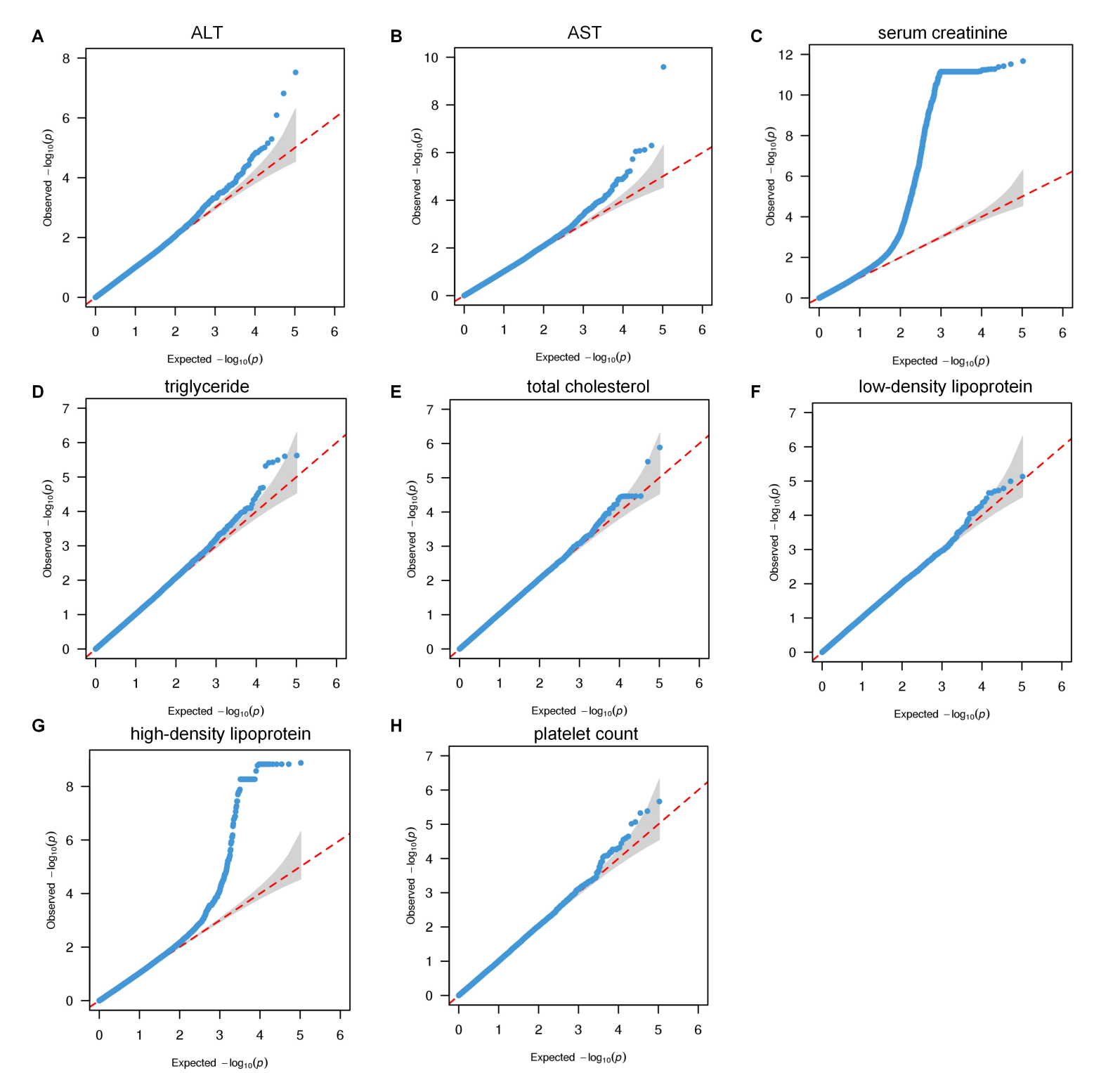


**Figure S3** Q-Q plot of genome-wide association

1. alanine aminotransferase (ALT). (B) aspartate aminotransferase (AST). (C) serum creatinine. (D) triglyceride. (E) total cholesterol. (F) low-density lipoprotein. (G) high-density lipoprotein. (H) platelet count.

**Table S1** Linear regression independent variable of covariates and indicators

| Dependent variable: PLT | | | | | | |
| --- | --- | --- | --- | --- | --- | --- |
| Model |  | Unstandardized Coefficients | | Standardized Coefficients | t | Significance |
|  |  | B | SE |  |  |  |
| 1 | constant | 106.966 | 35.448 |  | 3.018 | 0.003 |
|  | BMI | 6.45 | 1.555 | 0.299 | 4.146 | 5.25×10^-5^ |
| 2 | constant | 92.324 | 35.731 |  | 2.584 | 0.011 |
|  | BMI | 6.819 | 1.549 | 0.316 | 4.402 | 1.87×10^-5^ |
|  | SEX | 18.94 | 8.767 | 0.155 | 2.161 | 0.032 |
|  |  |  |  |  |  |  |
|  |  |  |  |  |  |  |
| Dependent variable: CREA | | | | | | |
| Model |  | Unstandardized Coefficients | | Standardized Coefficients | t | Significance |
|  |  | B | SE |  |  |  |
| 1 | constant | 81.235 | 0.662 |  | 122.658 | 0 |
|  | SEX | -21.222 | 1.166 | -0.597 | -18.204 | 3.13×10^-59^ |
| 2 | constant | 50.861 | 5.732 |  | 8.873 | 8.28×10^-18^ |
|  | SEX | -21.566 | 1.142 | -0.607 | -18.89 | 1.01×10^-62^ |
|  | BMI | 1.348 | 0.253 | 0.171 | 5.333 | 1.37×10^-7^ |
|  |  |  |  |  |  |  |
|  |  |  |  |  |  |  |
| Dependent variable: ALT | | | | | | |
| Model |  | Unstandardized Coefficients | | Standardized Coefficients | t | Significance |
|  |  | B | SE |  |  |  |
| 1 | constant | 18.863 | 0.45 |  | 41.93 | 4.55×10^-180^ |
|  | SEX | -6.316 | 0.792 | -0.31 | -7.976 | 7.76×10^-15^ |
| 2 | constant | -6.146 | 3.85 |  | -1.596 | 0.111 |
|  | SEX | -6.599 | 0.767 | -0.324 | -8.606 | 6.73×10^-17^ |
|  | BMI | 1.11 | 0.17 | 0.246 | 6.538 | 1.34×10^-10^ |
|  |  |  |  |  |  |  |
|  |  |  |  |  |  |  |
| Dependent variable: AST | | | | | | |
| Model |  | Unstandardized Coefficients | | Standardized Coefficients | t | Significance |
|  |  | B | SE |  |  |  |
| 1 | constant | 19.004 | 0.238 |  | 79.821 | 0 |
|  | SEX | -1.84 | 0.42 | -0.177 | -4.382 | 1.39×10^-5^ |
| 2 | constant | 11.449 | 2.087 |  | 5.486 | 6.08×10^-8^ |
|  | SEX | -1.928 | 0.416 | -0.185 | -4.631 | 4.48×10^-6^ |
|  | BMI | 0.335 | 0.092 | 0.146 | 3.643 | 2.93×10^-4^ |
|  |  |  |  |  |  |  |
|  |  |  |  |  |  |  |
|  |  |  |  |  |  |  |
|  |  |  |  |  |  |  |
|  |  |  |  |  |  |  |
| Dependent variable: TG | | | | | | |
| Model |  | Unstandardized Coefficients | | Standardized Coefficients | t | Significance |
|  |  | B | SE |  |  |  |
| 1 | constant | 0.479 | 0.074 |  | 6.492 | 1.96×10^-10^ |
|  | AGE | 0.021 | 0.002 | 0.337 | 8.217 | 1.63×10^-15^ |
| 2 | constant | -0.123 | 0.23 |  | -0.533 | 0.594 |
|  | AGE | 0.018 | 0.003 | 0.293 | 6.705 | 5.23×10^-11^ |
|  | BMI | 0.03 | 0.011 | 0.121 | 2.761 | 0.006 |
| 3 | constant | -0.133 | 0.229 |  | -0.583 | 0.56 |
|  | AGE | 0.019 | 0.003 | 0.308 | 6.986 | 8.62×10^-12^ |
|  | BMI | 0.031 | 0.011 | 0.124 | 2.851 | 0.005 |
|  | SEX | -0.102 | 0.045 | -0.094 | -2.283 | 0.023 |
|  |  |  |  |  |  |  |
|  |  |  |  |  |  |  |
| Dependent variable: TC | | | | | | |
| Model |  | Unstandardized Coefficients | | Standardized Coefficients | t | Significance |
|  |  | B | SE |  |  |  |
| 1 | constant | 3.652 | 0.112 |  | 32.617 | 2.43×10^-128^ |
|  | AGE | 0.023 | 0.004 | 0.26 | 6.181 | 1.28×10^-9^ |
| 2 | constant | 2.297 | 0.345 |  | 6.653 | 7.26×10^-11^ |
|  | AGE | 0.017 | 0.004 | 0.193 | 4.338 | 1.72×10^-5^ |
|  | BMI | 0.068 | 0.016 | 0.185 | 4.141 | 4.03×10^-5^ |
|  |  |  |  |  |  |  |
|  |  |  |  |  |  |  |
| Dependent variable: LDL | | | | | | |
| Model |  | Unstandardized Coefficients | | Standardized Coefficients | t | Significance |
|  |  | B | SE |  |  |  |
| 1 | constant | 0.52 | 0.331 |  | 1.573 | 0.117 |
|  | BMI | 0.087 | 0.015 | 0.284 | 5.903 | 7.65×10^-9^ |
| 2 | constant | 0.49 | 0.326 |  | 1.5 | 0.134 |
|  | BMI | 0.069 | 0.015 | 0.226 | 4.483 | 9.64×10^-6^ |
|  | AGE | 0.016 | 0.005 | 0.175 | 3.472 | 0.001 |
|  |  |  |  |  |  |  |
|  |  |  |  |  |  |  |
| Dependent variable: HDL | | | | | | |
| Model |  | Unstandardized Coefficients | | Standardized Coefficients | t | Significance |
|  |  | B | SE |  |  |  |
| 1 | constant | 1.314 | 0.016 |  | 80.041 | 4.75×10^-247^ |
|  | SEX | 0.161 | 0.03 | 0.26 | 5.358 | 1.43×10^-7^ |

**Table S2** Single nucleotide polymorphism correlated to ALT

| SNP | Chr | Position | Ref Allele | Min Allele | MAF | Bonferroni *p* value | FDR  *p* value | Gene | Position |
| --- | --- | --- | --- | --- | --- | --- | --- | --- | --- |
| rs146981251 | 1 | 1323965 | A | C | 0.012 | 3.01×10^-8^ | 0.003 | CCNL2 | intronic |
| rs117916372 | 12 | 114397634 | A | G | 0.032 | 1.53×10^-7^ | 0.008 | RBM19 | intronic |
| rs138264258 | 14 | 54879503 | C | T | 0.012 | 8.12×10^-7^ | 0.028 | CDKN3 | intronic |
| rs4594664 | 4 | 80977078 | G | T | 0.030 | 5.10×10^-6^ | 0.134 | ANTXR2 | intronic |
| rs145378993 | 1 | 1355796 | C | T | 0.011 | 7.04×10^-6^ | 0.148 | ANKRD65 | exonic |
| rs141659881 | 15 | 89694986 | G | A | 0.013 | 9.76×10^-6^ | 0.152 | ABHD2 | exonic |
| rs10232465 | 7 | 44065728 | G | A | 0.025 | 1.07×10^-5^ | 0.152 | POLR2J4 | intergenic |
| rs2248745 | 1 | 153509892 | A | G | 0.097 | 1.22×10^-5^ | 0.152 | S100A5 | exonic |
| rs3851 | 17 | 4686414 | A | C | 0.436 | 1.44×10^-5^ | 0.152 | TM4SF5 | exonic |
| rs34729309 | 2 | 177034121 | G | T | 0.033 | 1.45×10^-5^ | 0.152 | HOXD3 | exonic |
| rs12753531 | 1 | 226075608 | G | A | 0.019 | 1.68×10^-5^ | 0.160 | LEFTY1 | exonic |
| rs72711231 | 8 | 124658203 | T | C | 0.223 | 1.90×10^-5^ | 0.166 | KLHL38 | exonic |
| rs11869460 | 17 | 4686608 | G | A | 0.432 | 2.32×10^-5^ | 0.187 | TM4SF5 | downstream |
| rs17847749 | 1 | 204419139 | G | C | 0.012 | 2.57×10^-5^ | 0.192 | PIK3C2B | exonic |
| rs11546 | 14 | 56146357 | G | A | 0.017 | 3.63×10^-5^ | 0.246 | KTN1 | exonic |
| rs2274075 | 14 | 56096731 | A | G | 0.018 | 3.91×10^-5^ | 0.246 | KTN1 | exonic |
| rs9478857 | 6 | 151243467 | G | A | 0.023 | 4.11×10^-5^ | 0.246 | MTHFD1L | intronic |
| rs7972986 | 12 | 42481732 | G | A | 0.171 | 4.23×10^-5^ | 0.246 | GXYLT1 | exonic |
| rs181498922 | 8 | 117658968 | G | T | 0.013 | 4.61×10^-5^ | 0.254 | EIF3H | intronic |
| rs12973968 | 19 | 55501866 | G | C | 0.018 | 5.17×10^-5^ | 0.271 | NLRP2 | intronic |
| rs3752153 | 19 | 19822954 | C | T | 0.010 | 6.65×10^-5^ | 0.325 | ZNF14 | exonic |
| rs3730868 | 19 | 48660238 | C | T | 0.011 | 6.98×10^-5^ | 0.325 | LIG1 | intronic |
| rs495165 | 22 | 18844888 | A | G | 0.014 | 7.57×10^-5^ | 0.325 | LOC101929738 | intergenic |
| rs11667481 | 19 | 55497826 | G | A | 0.035 | 7.92×10^-5^ | 0.325 | NLRP2 | intronic |
| rs11228420 | 11 | 56216821 | G | A | 0.010 | 7.97×10^-5^ | 0.325 | OR5R1 | intergenic |
| rs28550886 | 6 | 3179987 | C | A | 0.013 | 8.35×10^-5^ | 0.325 | TUBB2A | intergenic |
| rs74053651 | 14 | 56125366 | C | T | 0.018 | 8.36×10^-5^ | 0.325 | KTN1 | intronic |
| rs16898691 | 8 | 124663987 | C | G | 0.235 | 9.53×10^-5^ | 0.345 | KLHL38 | exonic |
| rs12608926 | 19 | 33490488 | G | A | 0.025 | 9.54×10^-5^ | 0.345 | RHPN2 | intronic |

Chr: chromosome, Ref allele: reference allele, Min: minor allele, MAF: minor allele frequency, FDR: false discovery rate

**Table S3** Single nucleotide polymorphism correlated to AST

| SNP | Chr | Position | Ref Allele | Min Allele | MAF | Bonferroni *p* value | FDR  *p* value | Gene | Position |
| --- | --- | --- | --- | --- | --- | --- | --- | --- | --- |
| rs117916372 | 12 | 114397634 | A | G | 0.032 | 2.56×10^-10^ | 2.68×10^-5^ | RBM19 | intronic |
| rs532637 | 11 | 56468020 | T | C | 0.107 | 5.06×10^-7^ | 0.019 | OR9G1 | exonic |
| rs73168325 | 7 | 100612786 | G | T | 0.097 | 7.68×10^-7^ | 0.019 | MUC12 | upstream |
| rs12794886 | 11 | 62383715 | G | C | 0.101 | 8.45×10^-7^ | 0.019 | B3GAT3 | intronic |
| rs12359035 | 10 | 3208557 | A | G | 0.469 | 8.94×10^-7^ | 0.019 | PITRM1 | exonic |
| rs1851722 | 9 | 107361642 | G | A | 0.349 | 1.88×10^-6^ | 0.033 | OR13C5 | exonic |
| rs80216984 | 12 | 40899006 | C | A | 0.087 | 5.97×10^-6^ | 0.085 | MUC19 | intronic |
| rs4679392 | 3 | 124646594 | A | G | 0.069 | 6.53×10^-6^ | 0.085 | MUC13 | exonic |
| rs192861143 | 2 | 172305177 | C | T | 0.070 | 9.52×10^-6^ | 0.099 | DCAF17 | intronic |
| rs35065926 | 7 | 135375925 | A | C | 0.194 | 1.13×10^-5^ | 0.099 | SLC13A4 | intronic |
| rs78729591 | 11 | 56143795 | G | A | 0.096 | 1.32×10^-5^ | 0.099 | OR8U1 | exonic |
| rs202193035 | 11 | 56143803 | A | G | 0.096 | 1.32×10^-5^ | 0.099 | OR8U1 | exonic |
| rs200433243 | 11 | 56143804 | G | C | 0.096 | 1.32×10^-5^ | 0.099 | OR8U1 | exonic |
| rs201727202 | 11 | 56143807 | G | A | 0.096 | 1.32×10^-5^ | 0.099 | OR8U1 | exonic |
| rs17849408 | 12 | 21623179 | T | C | 0.094 | 1.54×10^-5^ | 0.108 | RECQL | exonic |
| rs201982103 | 11 | 56143810 | T | C | 0.092 | 2.16×10^-5^ | 0.133 | OR8U1 | exonic |
| rs111512467 | 11 | 56143816 | G | C | 0.092 | 2.16×10^-5^ | 0.133 | OR8U1 | exonic |
| rs6791801 | 3 | 45132577 | A | T | 0.201 | 2.40×10^-5^ | 0.140 | CDCP1 | intronic |
| rs4850122 | 2 | 132517068 | A | G | 0.147 | 2.66×10^-5^ | 0.147 | C2orf27A | intronic |
| rs17248 | 7 | 142231701 | T | G | 0.413 | 3.78×10^-5^ | 0.196 | TRBV10-1 | exonic |
| rs1879240 | 3 | 45132637 | T | C | 0.199 | 3.92×10^-5^ | 0.196 | CDCP1 | intronic |
| rs79033032 | 17 | 15584062 | A | G | 0.028 | 4.42×10^-5^ | 0.210 | TRIM16 | intronic |
| rs72711231 | 8 | 124658203 | T | C | 0.223 | 4.94×10^-5^ | 0.225 | KLHL38 | exonic |
| rs113921609 | 16 | 60392643 | G | A | 0.049 | 5.26×10^-5^ | 0.230 | LOC729159 | exonic |
| rs75055377 | 15 | 54336573 | C | T | 0.010 | 5.84×10^-5^ | 0.230 | UNC13C | intronic |
| rs56019945 | 6 | 108061325 | G | A | 0.043 | 5.90×10^-5^ | 0.230 | SCML4 | intronic |
| rs76876002 | 2 | 165811086 | G | A | 0.012 | 6.26×10^-5^ | 0.230 | LOC101929633 | intronic |
| rs117077996 | 2 | 165811682 | C | A | 0.012 | 6.26×10^-5^ | 0.230 | LOC101929633 | intronic |
| rs7635677 | 3 | 64554254 | C | T | 0.165 | 6.38×10^-5^ | 0.230 | LOC101929335 | intronic |
| rs17879464 | 19 | 36640642 | G | A | 0.014 | 7.96×10^-5^ | 0.272 | CAPNS1 | exonic |
| rs57186801 | 11 | 1628965 | G | A | 0.064 | 8.08×10^-5^ | 0.272 | KRTAP5-3 | exonic |
| rs4985162 | 16 | 15111218 | C | T | 0.369 | 8.45×10^-5^ | 0.272 | PDXDC1 | exonic |
| rs12272129 | 11 | 134177205 | G | T | 0.292 | 9.28×10^-5^ | 0.272 | GLB1L3 | intronic |
| rs3851 | 17 | 4686414 | A | C | 0.436 | 9.61×10^-5^ | 0.272 | TM4SF5 | exonic |
| rs7973815 | 12 | 100613964 | A | G | 0.019 | 9.80×10^-5^ | 0.272 | ACTR6 | intronic |

Chr: chromosome, Ref allele: reference allele, Min: minor allele, MAF: minor allele frequency, FDR: false discovery rate

**Table S3** Single nucleotide polymorphism correlated to triglyceride

| SNP | Chr | Position | Ref Allele | Min Allele | MAF | Bonferroni *p* value | FDR  *p* value | Gene | Position |
| --- | --- | --- | --- | --- | --- | --- | --- | --- | --- |
| rs140801520 | 20 | 50224040 | A | C | 0.017 | 2.38×10^-6^ | 0.079 | ATP9A | intronic |
| rs183750007 | 22 | 29630187 | G | C | 0.014 | 2.50×10^-6^ | 0.079 | EMID1 | exonic |
| rs78749212 | 12 | 65471485 | C | T | 0.016 | 3.22×10^-6^ | 0.079 | WIF1 | intronic |
| rs2234574 | 4 | 1985668 | G | A | 0.010 | 3.74×10^-6^ | 0.079 | NELFA | exonic |
| rs181284272 | 7 | 128506834 | C | T | 0.013 | 3.86×10^-6^ | 0.079 | LOC100130705 | exonic |
| rs182487183 | 9 | 127219919 | T | A | 0.012 | 4.80×10^-6^ | 0.082 | GPR144 | intronic |
| rs2293079 | 2 | 220100787 | C | T | 0.037 | 2.04×10^-5^ | 0.274 | ANKZF1 | exonic |
| rs2718131 | 7 | 138602916 | G | C | 0.110 | 2.13×10^-5^ | 0.274 | KIAA1549 | exonic |
| rs200263718 | 4 | 162577640 | A | G | 0.013 | 2.87×10^-5^ | 0.327 | FSTL5 | exonic |
| rs76017264 | 1 | 44126143 | C | T | 0.014 | 3.42×10^-5^ | 0.351 | KDM4A | intronic |
| rs117254261 | 1 | 151112158 | C | T | 0.016 | 4.23×10^-5^ | 0.395 | SEMA6C | exonic |
| rs11938463 | 4 | 1834422 | C | A | 0.016 | 4.65×10^-5^ | 0.399 | LETM1 | intronic |
| rs712833 | 17 | 74266322 | T | C | 0.046 | 6.19×10^-5^ | 0.461 | UBALD2 | exonic |
| rs59261129 | 12 | 49951377 | C | T | 0.012 | 8.01×10^-5^ | 0.461 | KCNH3 | exonic |
| rs1012874 | 12 | 50146950 | T | C | 0.012 | 8.01×10^-5^ | 0.461 | TMBIM6 | intronic |
| rs17123927 | 12 | 50149347 | A | G | 0.012 | 8.01×10^-5^ | 0.461 | TMBIM6 | intronic |
| rs10506287 | 12 | 50156640 | T | C | 0.012 | 8.01×10^-5^ | 0.461 | TMBIM6 | intronic |
| rs149461989 | 19 | 17186163 | A | G | 0.024 | 8.47×10^-5^ | 0.461 | HAUS8 | intronic |
| rs117820338 | 7 | 99703013 | A | C | 0.049 | 8.58×10^-5^ | 0.461 | AP4M1 | intronic |

Chr: chromosome, Ref allele: reference allele, Min: minor allele, MAF: minor allele frequency, FDR: false discovery rate

**Table S5** Single nucleotide polymorphism correlated to total cholesterol

| SNP | Chr | Position | Ref Allele | Min Allele | MAF | Bonferroni *p* value | FDR  *p* value | Gene | Position |
| --- | --- | --- | --- | --- | --- | --- | --- | --- | --- |
| rs4146708 | 1 | 248263626 | T | A | 0.137 | 1.31×10^-6^ | 0.134 | OR2L13 | UTR3 |
| rs41295946 | 1 | 248285906 | G | A | 0.123 | 3.39×10^-6^ | 0.174 | OR2M1P | exonic |
| rs41286118 | 13 | 76210890 | C | T | 0.105 | 3.43×10^-5^ | 0.386 | LMO7 | intronic |
| rs17258724 | 4 | 106924931 | G | A | 0.341 | 3.43×10^-5^ | 0.386 | LOC101929577 | exonic |
| rs7563561 | 2 | 234598991 | T | G | 0.269 | 3.46×10^-5^ | 0.386 | UGT1A10,UGT1A7,UGT1A8,UGT1A9 | intronic |
| rs7608175 | 2 | 234599089 | C | G | 0.269 | 3.46×10^-5^ | 0.386 | UGT1A10,UGT1A7,UGT1A8,UGT1A9 | intronic |
| rs1105880 | 2 | 234601965 | A | G | 0.269 | 3.46×10^-5^ | 0.386 | UGT1A6 | exonic |
| rs1105879 | 2 | 234602202 | A | C | 0.269 | 3.46×10^-5^ | 0.386 | UGT1A6 | exonic |
| rs6759892 | 2 | 234601669 | T | G | 0.268 | 3.54×10^-5^ | 0.386 | UGT1A6 | exonic |
| rs148575803 | 2 | 186673618 | C | A | 0.056 | 3.75×10^-5^ | 0.386 | FSIP2 | exonic |
| rs143400973 | 19 | 2717451 | G | A | 0.020 | 4.40×10^-5^ | 0.412 | DIRAS1 | exonic |
| rs10788986 | 1 | 55277430 | T | A | 0.047 | 5.89×10^-5^ | 0.454 | CIMAP2 | intronic |
| rs73141283 | 1 | 248309356 | A | G | 0.092 | 6.03×10^-5^ | 0.454 | OR2M5 | exonic |
| rs189647894 | 3 | 51470454 | C | A | 0.020 | 6.18×10^-5^ | 0.454 | VPRBP | intronic |
| rs188715061 | 2 | 70392120 | C | T | 0.011 | 7.69×10^-5^ | 0.478 | C2orf42 | intronic |
| rs9669474 | 12 | 118509347 | T | C | 0.203 | 7.70×10^-5^ | 0.478 | VSIG10 | intronic |
| rs3741298 | 11 | 116657561 | T | C | 0.422 | 8.05×10^-5^ | 0.478 | ZNF259 | intronic |
| rs186462828 | 3 | 9166190 | T | G | 0.022 | 8.37×10^-5^ | 0.478 | SRGAP3 | intronic |

Chr: chromosome, Ref allele: reference allele, Min: minor allele, MAF: minor allele frequency, FDR: false discovery rate

**Table S6** Single nucleotide polymorphism correlated to low density lipoprotein

| SNP | Chr | Position | Ref Allele | Min Allele | MAF | Bonferroni *p* value | FDR  *p* value | Gene | Position |
| --- | --- | --- | --- | --- | --- | --- | --- | --- | --- |
| rs10788986 | 1 | 55277430 | T | A | 0.062 | 7.42×10^-6^ | 0.333 | LEXM | intronic |
| rs41268345 | 1 | 247486213 | G | A | 0.071 | 1.01×10^-5^ | 0.333 | ZNF496 | intronic |
| rs117220350 | 3 | 15727638 | C | G | 0.014 | 1.64×10^-5^ | 0.333 | ANKRD28 | intronic |
| rs1982768 | 8 | 25898565 | A | C | 0.040 | 1.88×10^-5^ | 0.333 | EBF2 | intronic |
| rs2074087 | 16 | 16184232 | G | C | 0.194 | 1.98×10^-5^ | 0.333 | ABCC1 | intronic |
| rs12783379 | 10 | 20506326 | T | C | 0.229 | 2.22×10^-5^ | 0.333 | PLXDC2 | intronic |
| rs57946353 | 19 | 44977039 | A | G | 0.111 | 2.23×10^-5^ | 0.333 | ZNF229,ZNF180 | intergenic |
| rs28763868 | 2 | 170367099 | G | A | 0.011 | 3.18×10^-5^ | 0.414 | KLHL41 | exonic |
| rs4232870 | 17 | 74541129 | G | A | 0.258 | 3.96×10^-5^ | 0.443 | PRCD | UTR3 |
| rs9881057 | 3 | 86063065 | G | T | 0.214 | 4.25×10^-5^ | 0.443 | CADM2-AS1 | intronic |
| rs74354350 | 5 | 122522870 | G | A | 0.013 | 5.38×10^-5^ | 0.447 | PRDM6 | exonic |
| rs3817405 | 10 | 20506418 | G | A | 0.254 | 5.72×10^-5^ | 0.447 | PLXDC2 | exonic |
| rs36097019 | 4 | 152201053 | G | A | 0.349 | 6.29×10^-5^ | 0.447 | PRSS48 | exonic |
| rs142561549 | 7 | 63033547 | C | A | 0.086 | 6.39×10^-5^ | 0.447 | LOC100287834 | intergenic |
| rs76212882 | 5 | 35962873 | C | T | 0.072 | 7.58×10^-5^ | 0.447 | UGT3A1 | UTR3 |
| rs13126069 | 4 | 152212486 | C | T | 0.350 | 7.83×10^-5^ | 0.447 | PRSS48 | exonic |
| rs111637000 | 7 | 63033297 | C | T | 0.087 | 8.76×10^-5^ | 0.447 | LOC100287834 | intergenic |
| rs75415457 | 19 | 6376074 | C | T | 0.014 | 8.89×10^-5^ | 0.447 | PSPN | upstream |
| rs117006360 | 16 | 81174978 | A | G | 0.213 | 9.00×10^-5^ | 0.447 | PKD1L2 | exonic |

Chr: chromosome, Ref allele: reference allele, Min: minor allele, MAF: minor allele frequency, FDR: false discovery rate

**Table S7** Single nucleotide polymorphism correlated to high density lipoprotein

| SNP | Chr | Position | Ref Allele | Min Allele | MAF | Bonferroni *p* value | FDR  *p* value | Gene | Position |
| --- | --- | --- | --- | --- | --- | --- | --- | --- | --- |
| rs2761542 | 1 | 16973400 | G | A | 0.474 | 1.32×10^-9^ | 1.40×10^-5^ | MST1P2 | intronic |
| rs2799192 | 1 | 145018023 | G | T | 0.149 | 1.48×10^-9^ | 1.40×10^-5^ | PDE4DIP | intronic |
| rs75254397 | 7 | 100549414 | C | A | 0.149 | 1.48×10^-9^ | 1.40×10^-5^ | MUC3A | intronic |
| rs75547895 | 7 | 100549415 | A | T | 0.149 | 1.48×10^-9^ | 1.40×10^-5^ | MUC3A | intronic |
| rs75196671 | 7 | 100549421 | G | C | 0.149 | 1.48×10^-9^ | 1.40×10^-5^ | MUC3A | intronic |
| rs78470577 | 7 | 100549422 | A | T | 0.149 | 1.48×10^-9^ | 1.40×10^-5^ | MUC3A | intronic |
| rs78724937 | 7 | 100549426 | A | G | 0.149 | 1.48×10^-9^ | 1.40×10^-5^ | MUC3A | intronic |
| rs78097765 | 7 | 100612865 | C | T | 0.149 | 1.48×10^-9^ | 1.40×10^-5^ | MUC12 | upstream |
| rs74554402 | 7 | 100612869 | G | A | 0.149 | 1.48×10^-9^ | 1.40×10^-5^ | MUC12 | upstream |
| rs73168327 | 7 | 100612898 | T | C | 0.149 | 1.48×10^-9^ | 1.40×10^-5^ | MUC12 | upstream |
| rs79124801 | 11 | 48347144 | T | C | 0.149 | 1.48×10^-9^ | 1.40×10^-5^ | OR4C3 | exonic |
| rs73168325 | 7 | 100612786 | G | T | 0.146 | 1.65×10^-9^ | 1.44×10^-5^ | MUC12 | upstream |
| rs1964835 | 2 | 114387907 | C | T | 0.341 | 2.65×10^-9^ | 1.76×10^-5^ | RABL2A | intronic |
| rs80334520 | 11 | 56143556 | T | A | 0.145 | 5.39×10^-9^ | 1.76×10^-5^ | OR8U1 | exonic |
| rs74603674 | 11 | 56143559 | C | T | 0.145 | 5.39×10^-9^ | 1.76×10^-5^ | OR8U1 | exonic |
| rs75242425 | 11 | 56143561 | A | G | 0.145 | 5.39×10^-9^ | 1.76×10^-5^ | OR8U1 | exonic |
| rs80111030 | 11 | 56143562 | A | G | 0.145 | 5.39×10^-9^ | 1.76×10^-5^ | OR8U1 | exonic |
| rs77640279 | 11 | 56143564 | G | T | 0.145 | 5.39×10^-9^ | 1.76×10^-5^ | OR8U1 | exonic |
| rs4990122 | 11 | 56143696 | C | T | 0.145 | 5.39×10^-9^ | 1.76×10^-5^ | OR8U1 | exonic |
| rs4990121 | 11 | 56143699 | T | G | 0.145 | 5.39×10^-9^ | 1.76×10^-5^ | OR8U1 | exonic |
| rs10896309 | 11 | 56143716 | T | C | 0.145 | 5.39×10^-9^ | 1.76×10^-5^ | OR8U1 | exonic |
| rs10896310 | 11 | 56143717 | G | A | 0.145 | 5.39×10^-9^ | 1.76×10^-5^ | OR8U1 | exonic |
| rs12272349 | 11 | 56143723 | T | C | 0.145 | 5.39×10^-9^ | 1.76×10^-5^ | OR8U1 | exonic |
| rs77042014 | 11 | 56143725 | C | G | 0.145 | 5.39×10^-9^ | 1.76×10^-5^ | OR8U1 | exonic |
| rs55888197 | 11 | 56143729 | C | T | 0.145 | 5.39×10^-9^ | 1.76×10^-5^ | OR8U1 | exonic |
| rs55860603 | 11 | 56143730 | C | G | 0.145 | 5.39×10^-9^ | 1.76×10^-5^ | OR8U1 | exonic |
| rs79307272 | 11 | 56143762 | C | T | 0.145 | 5.39×10^-9^ | 1.76×10^-5^ | OR8U1 | exonic |
| rs79705206 | 11 | 56143767 | C | T | 0.145 | 5.39×10^-9^ | 1.76×10^-5^ | OR8U1 | exonic |
| rs78729591 | 11 | 56143795 | G | A | 0.145 | 5.39×10^-9^ | 1.76×10^-5^ | OR8U1 | exonic |
| rs202193035 | 11 | 56143803 | A | G | 0.145 | 5.39×10^-9^ | 1.76×10^-5^ | OR8U1 | exonic |
| rs200433243 | 11 | 56143804 | G | C | 0.145 | 5.39×10^-9^ | 1.76×10^-5^ | OR8U1 | exonic |
| rs201727202 | 11 | 56143807 | G | A | 0.145 | 5.39×10^-9^ | 1.76×10^-5^ | OR8U1 | exonic |
| rs555507590 | 16 | 33965666 | G | A | 0.145 | 1.29×10^-8^ | 4.08×10^-5^ | LINC00273 | intergenic |
| rs3883945 | 21 | 10862599 | C | T | 0.160 | 1.51×10^-8^ | 4.63×10^-5^ | LOC101927345 | intergenic |
| rs12794886 | 11 | 62383715 | G | C | 0.153 | 1.55×10^-8^ | 4.63×10^-5^ | B3GAT3 | intronic |
| rs532637 | 11 | 56468020 | T | C | 0.161 | 1.72×10^-8^ | 5.00×10^-5^ | OR9G1 | exonic |
| rs793079 | 2 | 114391721 | T | C | 0.300 | 2.00×10^-8^ | 5.64×10^-5^ | RABL2A | intronic |
| rs201982103 | 11 | 56143810 | T | C | 0.139 | 3.56×10^-8^ | 9.52×10^-5^ | OR8U1 | exonic |
| rs111512467 | 11 | 56143816 | G | C | 0.139 | 3.56×10^-8^ | 9.52×10^-5^ | OR8U1 | exonic |
| rs11726970 | 4 | 174537221 | T | C | 0.223 | 5.31×10^-8^ | 1.38×10^-4^ | HAND2-AS1 | intergenic |
| rs814130 | 3 | 127410959 | G | T | 0.311 | 6.19×10^-8^ | 1.57×10^-4^ | MGLL | UTR3 |
| rs80216984 | 12 | 40899006 | C | A | 0.131 | 8.74×10^-8^ | 2.17×10^-4^ | MUC19 | intronic |
| rs17248 | 7 | 142231701 | T | G | 0.499 | 1.30×10^-7^ | 3.14×10^-4^ | TRBV10-1 | exonic |
| rs144934775 | 9 | 99114231 | C | G | 0.011 | 1.32×10^-7^ | 3.14×10^-4^ | SLC35D2 | intronic |
| rs58308167 | 14 | 106866565 | C | A | 0.126 | 1.53×10^-7^ | 3.55×10^-4^ | IGHV3-38 | exonic |
| rs2288206 | 2 | 152417064 | G | A | 0.097 | 1.64×10^-7^ | 3.72×10^-4^ | NEB | intronic |
| rs28472548 | 19 | 40368395 | C | T | 0.458 | 1.74×10^-7^ | 3.85×10^-4^ | FCGBP | exonic |
| rs2314343 | 23 | 153048657 | A | C | 0.146 | 2.31×10^-7^ | 5.02×10^-4^ | SRPK3 | intergenic |
| rs9921162 | 16 | 15095675 | C | A | 0.125 | 2.47×10^-7^ | 5.27×10^-4^ | PDXDC1 | exonic |
| rs11549900 | 16 | 15098047 | C | G | 0.126 | 3.07×10^-7^ | 6.40×10^-4^ | PDXDC1 | exonic |
| rs3848300 | 16 | 55862605 | G | A | 0.409 | 6.55×10^-7^ | 1.34×10^-3^ | CES1 | intronic |
| rs11147976 | 13 | 21729252 | T | C | 0.055 | 7.82×10^-7^ | 1.54×10^-3^ | SKA3 | exonic |
| rs11147977 | 13 | 21729267 | G | A | 0.055 | 7.82×10^-7^ | 1.54×10^-3^ | SKA3 | exonic |
| rs7509970 | 21 | 45999716 | T | C | 0.064 | 1.16×10^-7^ | 2.21×10^-3^ | KRTAP10-5 | exonic |
| rs117501643 | 14 | 22978364 | C | G | 0.010 | 1.16×10^-7^ | 2.21×10^-3^ | TRAJ32 | exonic |
| rs17855271 | 24 | 21154569 | A | G | 0.413 | 1.19×10^-6^ | 2.21×10^-3^ | TTTY14 | intergenic |
| rs57946353 | 19 | 44977039 | A | G | 0.111 | 1.38×10^-6^ | 2.52×10^-3^ | ZNF229 | intergenic |
| rs4729627 | 7 | 100550428 | C | A | 0.120 | 2.26×10^-6^ | 4.06×10^-3^ | MUC3A | exonic |
| rs2272896 | 16 | 836992 | G | C | 0.093 | 2.55×10^-6^ | 4.52×10^-3^ | RPUSD1 | intronic |
| rs10001599 | 4 | 39918636 | A | T | 0.327 | 3.69×10^-6^ | 6.42×10^-3^ | PDS5A | intronic |
| rs4916129 | 1 | 248487300 | C | T | 0.341 | 4.08×10^-6^ | 6.99×10^-3^ | OR2M7 | exonic |
| rs4983661 | 15 | 20467100 | A | G | 0.123 | 4.63×10^-6^ | 7.74×10^-3^ | CHEK2P2 | intergenic |
| rs11645376 | 16 | 89912281 | G | C | 0.071 | 4.67×10^-6^ | 7.74×10^-3^ | SPIRE2 | intronic |
| rs2301627 | 17 | 48153924 | T | C | 0.263 | 5.53×10^-6^ | 9.00×10^-3^ | ITGA3 | intronic |
| rs17091582 | 3 | 195804002 | C | A | 0.052 | 5.69×10^-6^ | 9.00×10^-3^ | TFRC | intronic |
| rs17091585 | 3 | 195804060 | C | A | 0.052 | 5.69×10^-6^ | 9.00×10^-3^ | TFRC | intronic |
| rs367608484 | 7 | 142008529 | C | G | 0.088 | 6.03×10^-6^ | 9.25×10^-3^ | TRBV3-1 | upstream |
| rs373709044 | 7 | 142008542 | A | G | 0.088 | 6.03×10^-6^ | 9.25×10^-3^ | TRBV3-1 | upstream |
| rs7503829 | 17 | 36638255 | C | T | 0.304 | 7.52×10^-6^ | 0.011 | ARHGAP23 | intronic |
| rs11132309 | 4 | 186382206 | A | G | 0.084 | 8.20×10^-6^ | 0.012 | CCDC110 | exonic |
| rs2688542 | 3 | 195452951 | G | C | 0.496 | 9.86×10^-6^ | 0.015 | MUC20 | exonic |
| rs7121646 | 11 | 800485 | T | A | 0.083 | 1.11×10^-5^ | 0.016 | PIDD | intronic |
| rs3851873 | 1 | 144854597 | T | C | 0.419 | 1.31×10^-5^ | 0.019 | PDE4DIP | exonic |
| rs113026716 | 23 | 2836373 | A | G | 0.106 | 1.38×10^-5^ | 0.019 | ARSD | intergenic |
| rs10788986 | 1 | 55277430 | T | A | 0.062 | 1.48×10^-5^ | 0.021 | C1orf177 | intronic |
| rs7915504 | 10 | 111440719 | T | A | 0.069 | 1.54×10^-5^ | 0.021 | LOC101927573 | intergenic |
| rs76599809 | 8 | 27143737 | C | T | 0.043 | 1.63×10^-5^ | 0.022 | TRIM35 | UTR3 |
| rs17846974 | 2 | 241534092 | C | T | 0.038 | 1.78×10^-5^ | 0.024 | CAPN10 | exonic |
| rs12792259 | 11 | 22214903 | G | C | 0.380 | 1.84×10^-5^ | 0.024 | ANO5 | exonic |
| rs34906910 | 6 | 34003314 | A | T | 0.304 | 1.85×10^-5^ | 0.024 | GRM4 | intronic |
| rs73632972 | 23 | 2835964 | G | A | 0.107 | 1.86×10^-5^ | 0.024 | ARSD | intergenic |
| rs4073890 | 2 | 242680366 | A | G | 0.126 | 1.90×10^-5^ | 0.024 | D2HGDH | intronic |
| rs10425763 | 19 | 22155918 | T | C | 0.349 | 1.96×10^-5^ | 0.025 | ZNF208 | exonic |
| rs2405599 | 1 | 47611598 | T | C | 0.401 | 2.15×10^-5^ | 0.027 | CYP4A22 | exonic |
| rs3829069 | 9 | 2056563 | G | A | 0.332 | 2.29×10^-5^ | 0.028 | SMARCA2 | intronic |
| rs6498535 | 16 | 15092116 | C | T | 0.073 | 2.70×10^-5^ | 0.033 | PDXDC1 | intronic |
| rs57186801 | 11 | 1628965 | G | A | 0.096 | 2.80×10^-5^ | 0.034 | KRTAP5-3 | exonic |
| rs6974722 | 7 | 107738836 | T | C | 0.062 | 2.91×10^-5^ | 0.035 | LAMB4 | intronic |
| rs80095370 | 16 | 5037523 | C | G | 0.331 | 3.14×10^-5^ | 0.037 | SEC14L5 | intronic |
| rs2074535 | 12 | 48132889 | G | A | 0.117 | 3.24×10^-5^ | 0.037 | RAPGEF3 | intronic |
| rs9610445 | 22 | 36597744 | A | C | 0.048 | 3.25×10^-5^ | 0.037 | APOL4 | splicing |
| rs1255720 | 14 | 64009521 | A | G | 0.278 | 3.31×10^-5^ | 0.038 | PPP2R5E | UTR5 |
| rs375565405 | 15 | 85788358 | G | A | 0.185 | 3.47×10^-5^ | 0.039 | GOLGA6L3 | intronic |
| rs3064782 | 2 | 234740798 | T | A | 0.379 | 3.71×10^-5^ | 0.041 | MROH2A | intronic |
| rs3817918 | 1 | 6500919 | T | C | 0.117 | 3.88×10^-5^ | 0.043 | ESPN | intronic |
| rs35388621 | 17 | 36654005 | C | T | 0.019 | 3.93×10^-5^ | 0.043 | ARHGAP23 | intronic |
| rs1544445 | 7 | 149516168 | C | T | 0.088 | 3.98×10^-5^ | 0.043 | SSPO | intronic |
| rs12441468 | 15 | 22383064 | C | A | 0.126 | 4.29×10^-5^ | 0.046 | OR4N4 | exonic |
| rs62481928 | 7 | 128292780 | G | A | 0.256 | 4.36×10^-5^ | 0.046 | LINC01000 | exonic |
| rs3739580 | 9 | 2060768 | T | C | 0.299 | 4.61×10^-5^ | 0.048 | SMARCA2 | intronic |
| rs17849408 | 12 | 21623179 | T | C | 0.083 | 5.21×10^-5^ | 0.053 | RECQL | exonic |
| rs2072869 | 22 | 25714218 | A | T | 0.288 | 5.24×10^-5^ | 0.053 | IGLL3P | upstream |
| rs361402 | 7 | 142045897 | C | T | 0.146 | 5.25×10^-5^ | 0.053 | TRBV4-2 | downstream |
| rs9292078 | 5 | 54320700 | C | T | 0.418 | 5.92×10^-5^ | 0.059 | GZMK | intronic |
| rs1268065889 | 19 | 9009427 | A | G | 0.151 | 6.07×10^-5^ | 0.060 | MUC16 | intronic |
| rs77532430 | 17 | 27896307 | C | T | 0.010 | 6.39×10^-5^ | 0.063 | TP53I13 | intronic |
| rs12974755 | 19 | 12502787 | G | A | 0.024 | 7.20×10^-5^ | 0.070 | ZNF799 | exonic |
| rs3738339 | 1 | 220205704 | T | A | 0.097 | 7.65×10^-5^ | 0.074 | EPRS | intronic |
| rs12600401 | 17 | 41465867 | A | G | 0.228 | 8.13×10^-5^ | 0.077 | LINC00910 | exonic |
| rs114097201 | 7 | 76751301 | C | A | 0.045 | 8.17×10^-5^ | 0.077 | CCDC146 | upstream |
| rs7311413 | 12 | 80878174 | T | A | 0.306 | 8.18×10^-5^ | 0.077 | PTPRQ | intronic |
| rs4963512 | 12 | 6942823 | G | A | 0.044 | 8.31×10^-5^ | 0.077 | LEPREL2 | intronic |
| rs559754 | 15 | 22383066 | A | G | 0.412 | 9.27×10^-5^ | 0.086 | OR4N4 | exonic |
| rs2466613 | 11 | 124096247 | G | A | 0.115 | 9.53×10^-5^ | 0.087 | OR8G2 | exonic |
| rs2289952 | 2 | 101644316 | A | C | 0.013 | 9.65×10^-5^ | 0.087 | TBC1D8 | intronic |
| rs2303930 | 7 | 150761548 | A | G | 0.092 | 9.72×10^-5^ | 0.087 | SLC4A2 | intronic |

Chr: chromosome, Ref allele: reference allele, Min: minor allele, MAF: minor allele frequency, FDR: false discovery rate

**Table S8** Genetic Variants detected in both discovery and replication cohorts

| **Indicator** | **SNP** | **Gene** | **Ref Allele** | **Min Allele** | **Discovery Cohort** | | **Replication Cohort** | |
| --- | --- | --- | --- | --- | --- | --- | --- | --- |
|  |  |  |  |  | **MAF** | ***p* value** | **MAF** | ***p* value** |
| ALT | rs3851 | *TM4SF5* | A | C | 0.436 | 1.44×10^-5^ | 0.381 | 0.041 |
| AST | rs3851 | *TM4SF5* | A | C | 0.436 | 9.61×10^-5^ | 0.381 | 0.0031 |
| TG | rs712833 | *UBALD2* | T | C | 0.046 | 6.19×10^-5^ | 0.050 | 0.019 |
| HDL | rs3739580 | *SMARCA2* | T | C | 0.299 | 4.61×10^-5^ | 0.287 | 0.033 |
| CREA | rs80068592 | *FCGBP* | G | A | 0.029 | 5.72×10^-6^ | 0.088 | 2.23×10^-4^ |
| CREA | rs140898464 | *KRTAP4-11* | A | C | 0.026 | 3.09×10^-5^ | 0.011 | 0.0055 |
| CREA | rs61734664 | *ATP10B* | G | A | 0.013 | 7.16×10^-5^ | 0.019 | 0.010 |

Ref allele: reference allele, Min: minor allele, MAF: minor allele frequency

**Table S9** Single nucleotide polymorphism correlated to serum creatinine

| SNP | Chr | Position | Ref Allele | Min Allele | MAF | Bonferroni *p* value | FDR  *p* value | Gene | Position |
| --- | --- | --- | --- | --- | --- | --- | --- | --- | --- |
| rs62556711 | 9 | 45730241 | C | T | 0.063 | 2.13×10^-12^ | 8.24×10^-10^ | FAM27A,  FAM27E2 | intergenic |
| rs373513100 | 20 | 29623263 | C | T | 0.057 | 3.01×10^-12^ | 8.24×10^-10^ | FRG1B | intronic |
| rs531659 | 22 | 18769228 | G | A | 0.065 | 3.77×10^-12^ | 8.24×10^-10^ | GGT3P | exonic |
| rs2688538 | 3 | 195345953 | C | T | 0.059 | 4.15×10^-12^ | 8.24×10^-10^ | APOD,SDHAP2 | intergenic |
| rs5013161 | 10 | 127585240 | G | A | 0.054 | 5.29×10^-12^ | 8.24×10^-10^ | FANK1 | intronic |
| rs3901679 | 1 | 16901668 | T | C | 0.055 | 5.31×10^-12^ | 8.24×10^-10^ | NBPF1 | exonic |
| rs11727824 | 4 | 190874167 | G | A | 0.058 | 5.43×10^-12^ | 8.24×10^-10^ | FRG1 | intronic |
| rs58679574 | 1 | 16974511 | G | A | 0.059 | 5.68×10^-12^ | 8.24×10^-10^ | MST1P2 | exonic |
| rs76964780 | 11 | 48346535 | G | C | 0.059 | 5.78×10^-12^ | 8.24×10^-10^ | OR4C3 | exonic |
| rs10907850 | 9 | 66457588 | T | C | 0.058 | 5.81×10^-12^ | 8.24×10^-10^ | LOC101928301,LOC100132249 | intergenic |
| rs71443637 | 12 | 11244194 | T | C | 0.058 | 6.68×10^-12^ | 8.51×10^-10^ | TAS2R43 | exonic |
| rs2681142 | 8 | 12423373 | T | C | 0.053 | 6.80×10^-12^ | 8.56×10^-10^ | LOC100506990,LOC729732 | intronic |
| rs74588241 | 7 | 100549505 | T | C | 0.059 | 7.08×10^-12^ | 8.64×10^-10^ | MUC3A | exonic |
| rs61946465 | 13 | 19778557 | T | C | 0.049 | 7.15×10^-12^ | 8.69×10^-10^ | LOC101928697,ANKRD26P3 | intergenic |
| rs61841515 | 10 | 47151642 | C | T | 0.061 | 7.44×10^-12^ | 8.97×10^-10^ | LINC00842 | upstream |
| rs75563569 | 12 | 40899052 | A | C | 0.054 | 7.48×10^-12^ | 8.97×10^-10^ | MUC19 | intronic |
| rs2992339 | 1 | 16890644 | C | T | 0.056 | 7.82×10^-12^ | 8.97×10^-10^ | NBPF1 | exonic |
| rs5013162 | 10 | 127585263 | C | T | 0.056 | 8.55×10^-12^ | 8.97×10^-10^ | FANK1 | intronic |
| rs1360574 | 1 | 16972067 | C | T | 0.059 | 8.55×10^-12^ | 1.06×10^-9^ | LOC101927663,MST1P2 | upstream |
| rs559507 | 1 | 17023143 | C | T | 0.059 | 8.55×10^-12^ | 1.09×10^-9^ | ESPNP | exonic |
| rs2994081 | 1 | 12942289 | C | A | 0.059 | 8.58×10^-12^ | 1.09×10^-9^ | PRAMEF4 | intronic |
| rs36101709 | 11 | 1017783 | A | G | 0.058 | 9.99×10^-12^ | 1.13×10^-9^ | MUC6 | exonic |
| rs209032 | 21 | 11058349 | T | G | 0.059 | 1.09×10^-11^ | 1.14×10^-9^ | BAGE2 | intronic |
| rs11204658 | 10 | 46999484 | G | T | 0.059 | 1.17×10^-11^ | 1.14×10^-9^ | GPRIN2 | exonic |
| 24_13489493 | 24 | 13489493 | C | A | 0.058 | 1.19×10^-11^ | 1.22×10^-9^ | NA | intergenic |
| rs2994080 | 1 | 12942306 | C | T | 0.051 | 1.26×10^-11^ | 1.22×10^-9^ | PRAMEF4 | intronic |
| rs9728933 | 1 | 16907871 | C | T | 0.059 | 1.27×10^-11^ | 1.26×10^-9^ | NBPF1 | intronic |
| rs568699516 | 16 | 33965595 | C | A | 0.059 | 1.29×10^-11^ | 1.29×10^-9^ | LINC00273,LOC100505948 | intergenic |
| rs3820001 | 1 | 1582961 | G | C | 0.052 | 1.33×10^-11^ | 1.29×10^-9^ | CDK11B | intronic |
| rs1054924 | 21 | 14424092 | T | C | 0.064 | 1.37×10^-11^ | 1.29×10^-9^ | ANKRD30BP2 | intronic |
| rs6087171 | 20 | 29630695 | C | A | 0.057 | 1.45×10^-11^ | 1.29×10^-9^ | FRG1B | intronic |
| rs61779367 | 1 | 13001312 | C | G | 0.064 | 1.48×10^-11^ | 1.41×10^-9^ | PRAMEF6 | exonic |
| 24_13489418 | 24 | 13489418 | C | G | 0.058 | 1.84×10^-11^ | 1.45×10^-9^ | NA | intergenic |
| 24_13489427 | 24 | 13489427 | G | A | 0.058 | 1.84×10^-11^ | 1.55×10^-9^ | NA | intergenic |
| rs61814640 | 1 | 145301697 | C | T | 0.058 | 1.87×10^-11^ | 1.66×10^-9^ | NBPF10 | intronic |
| 24_13489415 | 24 | 13489415 | G | T | 0.059 | 1.88×10^-11^ | 1.66×10^-9^ | NA | intergenic |
| 24_13489446 | 24 | 13489446 | G | A | 0.059 | 1.88×10^-11^ | 1.86×10^-9^ | NA | intergenic |
| 24_13489482 | 24 | 13489482 | C | T | 0.059 | 1.88×10^-11^ | 1.86×10^-9^ | NA | intergenic |
| 24_13489491 | 24 | 13489491 | G | T | 0.059 | 1.88×10^-11^ | 1.86×10^-9^ | NA | intergenic |
| rs6818201 | 4 | 144824273 | C | T | 0.062 | 2.00×10^-11^ | 1.86×10^-9^ | GYPE | intronic |
| 24_13489465 | 24 | 13489465 | C | G | 0.058 | 2.10×10^-11^ | 1.93×10^-9^ | NA | intergenic |
| rs57251344 | 3 | 195701388 | G | A | 0.054 | 2.14×10^-11^ | 1.93×10^-9^ | SDHAP1 | exonic |
| rs60183692 | 3 | 195701389 | C | A | 0.054 | 2.14×10^-11^ | 2.08×10^-9^ | SDHAP1 | exonic |
| rs1057857 | 1 | 16383742 | C | G | 0.059 | 2.41×10^-11^ | 2.33×10^-9^ | CLCNKB | UTR3 |
| rs200168540 | 10 | 88988115 | G | A | 0.056 | 2.52×10^-11^ | 2.60×10^-9^ | NUTM2A | exonic |
| rs11260887 | 1 | 17023110 | G | A | 0.054 | 2.57×10^-11^ | 2.60×10^-9^ | ESPNP | exonic |
| rs796099497 | 9 | 68372409 | G | A | 0.059 | 2.62×10^-11^ | 2.60×10^-9^ | LOC644249,LINC00537 | intergenic |
| rs11260921 | 1 | 17084510 | G | A | 0.057 | 2.78×10^-11^ | 2.60×10^-9^ | MST1L | exonic |
| rs3868031 | 1 | 16893071 | T | G | 0.053 | 2.86×10^-11^ | 2.71×10^-9^ | NBPF1 | intronic |
| rs143647724 | 9 | 45730202 | G | A | 0.059 | 2.87×10^-11^ | 3.01×10^-9^ | FAM27A,FAM27E2 | intergenic |
| rs61790276 | 1 | 120611758 | G | A | 0.058 | 2.92×10^-11^ | 3.12×10^-9^ | NOTCH2 | intronic |
| rs10910778 | 1 | 145273302 | A | G | 0.058 | 2.92×10^-11^ | 3.12×10^-9^ | NOTCH2NL | exonic |
| rs12120756 | 1 | 145281613 | C | A | 0.058 | 2.92×10^-11^ | 3.14×10^-9^ | NOTCH2NL | exonic |
| rs62485525 | 7 | 144060286 | A | G | 0.056 | 3.06×10^-11^ | 3.22×10^-9^ | ARHGEF5 | exonic |
| rs1799887 | 7 | 142498687 | T | C | 0.063 | 3.09×10^-11^ | 3.22×10^-9^ | TRBC2 | upstream |
| rs74366995 | 9 | 68372407 | T | C | 0.059 | 3.09×10^-11^ | 3.22×10^-9^ | LOC644249,LINC00537 | intergenic |
| rs1057854 | 1 | 16383682 | G | C | 0.058 | 3.12×10^-11^ | 3.22×10^-9^ | CLCNKB | UTR3 |
| 24_13489402 | 24 | 13489402 | T | G | 0.056 | 3.81×10^-11^ | 3.22×10^-9^ | NA | intergenic |
| rs6663523 | 1 | 145296478 | G | T | 0.064 | 3.93×10^-11^ | 3.36×10^-9^ | NBPF10 | exonic |
| rs2009433 | 22 | 23247082 | C | G | 0.055 | 3.94×10^-11^ | 3.76×10^-9^ | IGLJ3 | upstream |
| rs4291472 | 1 | 21751214 | G | A | 0.063 | 4.06×10^-11^ | 3.79×10^-9^ | ECE1,  NBPF3 | intergenic |
| 24_13489469 | 24 | 13489469 | G | A | 0.059 | 4.34×10^-11^ | 3.81×10^-9^ | NA | intergenic |
| rs7535915 | 1 | 168025628 | G | A | 0.054 | 4.99×10^-11^ | 4.27×10^-9^ | DCAF6 | intronic |
| rs142201186 | 15 | 34849011 | C | T | 0.056 | 5.00×10^-11^ | 4.27×10^-9^ | GOLGA8B | intronic |
| rs28503658 | 1 | 16974666 | C | T | 0.058 | 5.20×10^-11^ | 4.27×10^-9^ | MST1P2 | exonic |
| rs2444187 | 15 | 20450242 | G | C | 0.052 | 5.81×10^-11^ | 4.27×10^-9^ | NONE,  CHEK2P2 | intergenic |
| rs80221306 | 1 | 152278555 | T | C | 0.042 | 6.07×10^-11^ | 4.27×10^-9^ | FLG | exonic |
| rs3887623 | 21 | 9907416 | C | T | 0.069 | 6.26×10^-11^ | 4.31×10^-9^ | TEKT4P2 | exonic |
| rs75707116 | 9 | 68372401 | C | T | 0.058 | 7.11×10^-11^ | 4.76×10^-9^ | LOC644249,LINC00537 | intergenic |
| 24_13489519 | 24 | 13489519 | A | T | 0.057 | 7.84×10^-11^ | 5.33×10^-9^ | NA | intergenic |
| rs758459500 | 11 | 65270602 | G | A | 0.025 | 9.11×10^-11^ | 6.64×10^-9^ | MALAT1 | exonic |
| rs3927729 | 1 | 145281408 | C | T | 0.056 | 9.32×10^-11^ | 6.95×10^-9^ | NOTCH2NL | exonic |
| rs28735758 | 9 | 41954644 | G | A | 0.055 | 9.52×10^-11^ | 7.22×10^-9^ | MGC21881 | exonic |
| rs430037 | 3 | 195511142 | T | C | 0.051 | 1.05×10^-10^ | 7.41×10^-9^ | MUC4 | exonic |
| rs79282413 | 3 | 195711016 | G | A | 0.054 | 1.15×10^-10^ | 7.41×10^-9^ | SDHAP1 | exonic |
| rs28406689 | 9 | 41954658 | G | A | 0.054 | 1.23×10^-10^ | 7.72×10^-9^ | MGC21881 | intronic |
| rs28758397 | 9 | 41954665 | C | A | 0.054 | 1.23×10^-10^ | 7.73×10^-9^ | MGC21881 | intronic |
| rs11685929 | 2 | 74649921 | C | A | 0.053 | 1.23×10^-10^ | 8.08×10^-9^ | WDR54 | intronic |
| rs10422078 | 19 | 4511278 | C | T | 0.053 | 1.41×10^-10^ | 9.37×10^-9^ | PLIN4 | exonic |
| rs80238130 | 19 | 4511283 | C | T | 0.053 | 1.41×10^-10^ | 9.37×10^-9^ | PLIN4 | exonic |
| rs10422854 | 19 | 4511284 | A | G | 0.053 | 1.41×10^-10^ | 9.37×10^-9^ | PLIN4 | exonic |
| rs200077789 | 3 | 197846736 | G | C | 0.051 | 1.44×10^-10^ | 9.37×10^-9^ | ANKRD18DP,FAM157A | intergenic |
| rs2072923 | 1 | 1599888 | A | G | 0.065 | 1.50×10^-10^ | 1.00×10^-8^ | SLC35E2B | exonic |
| rs61806690 | 1 | 121116688 | A | G | 0.060 | 1.52×10^-10^ | 1.03×10^-8^ | SRGAP2C | exonic |
| rs35732993 | 7 | 5770330 | T | C | 0.054 | 1.85×10^-10^ | 1.04×10^-8^ | RNF216 | intronic |
| rs652423 | 12 | 52844265 | T | C | 0.046 | 1.88×10^-10^ | 1.04×10^-8^ | KRT6B | exonic |
| rs6682375 | 1 | 14907 | A | G | 0.054 | 1.93×10^-10^ | 1.15×10^-8^ | WASH7P | intronic |
| rs6682385 | 1 | 14930 | A | G | 0.054 | 1.93×10^-10^ | 1.21×10^-8^ | WASH7P | intronic |
| rs3893071 | 9 | 34834494 | C | T | 0.054 | 2.05×10^-10^ | 1.34×10^-8^ | FAM205B | exonic |
| rs113593823 | 16 | 1277609 | A | G | 0.051 | 2.06×10^-10^ | 1.35×10^-8^ | TPSB2 | downstream |
| rs201789369 | 16 | 1277615 | A | G | 0.051 | 2.06×10^-10^ | 1.39×10^-8^ | TPSB2 | downstream |
| rs200060197 | 16 | 1277625 | C | T | 0.051 | 2.06×10^-10^ | 1.51×10^-8^ | TPSB2 | downstream |
| rs201380817 | 16 | 1277626 | C | G | 0.051 | 2.06×10^-10^ | 1.51×10^-8^ | TPSB2 | downstream |
| rs139488657 | 14 | 106235567 | G | A | 0.049 | 2.14×10^-10^ | 1.51×10^-8^ | IGHG3 | downstream |
| rs4042058 | 14 | 106235569 | C | G | 0.049 | 2.14×10^-10^ | 1.53×10^-8^ | IGHG3 | downstream |
| rs183684706 | 7 | 151927025 | A | G | 0.053 | 2.14×10^-10^ | 1.53×10^-8^ | KMT2C | exonic |
| rs1250973 | 10 | 81995007 | A | G | 0.045 | 2.25×10^-10^ | 1.53×10^-8^ | LINC00857,LOC100130698 | intergenic |
| rs612736 | 1 | 16903780 | C | T | 0.051 | 2.33×10^-10^ | 1.55×10^-8^ | NBPF1 | intronic |
| rs672182 | 8 | 12453555 | T | C | 0.052 | 2.55×10^-10^ | 1.55×10^-8^ | LOC729732 | exonic |
| rs200533443 | 9 | 45730207 | G | T | 0.047 | 2.60×10^-10^ | 1.55×10^-8^ | FAM27A,FAM27E2 | intergenic |
| rs71540915 | 7 | 100550282 | C | T | 0.046 | 2.78×10^-10^ | 1.55×10^-8^ | MUC3A | exonic |
| rs78054189 | 7 | 100550285 | C | T | 0.046 | 2.78×10^-10^ | 1.66×10^-8^ | MUC3A | exonic |
| rs370230558 | 7 | 100550286 | T | G | 0.046 | 2.78×10^-10^ | 1.68×10^-8^ | MUC3A | exonic |
| rs79792138 | 1 | 13052871 | C | T | 0.057 | 3.02×10^-10^ | 1.79×10^-8^ | PRAMEF22,PRAMEF23 | intergenic |
| rs202237204 | 9 | 45730195 | T | C | 0.045 | 3.43×10^-10^ | 1.79×10^-8^ | FAM27A,FAM27E2 | intergenic |
| rs112500726 | 2 | 132044787 | G | A | 0.049 | 3.46×10^-10^ | 2.41×10^-8^ | LOC440910 | intronic |
| rs73126218 | 3 | 10088343 | A | G | 0.052 | 3.50×10^-10^ | 2.46×10^-8^ | FANCD2 | exonic |
| rs7428473 | 3 | 180679156 | G | A | 0.052 | 4.02×10^-10^ | 2.60×10^-8^ | FXR1 | intronic |
| rs10430152 | 1 | 142667306 | C | T | 0.057 | 4.15×10^-10^ | 2.61×10^-8^ | MIR3118-1 | exonic |
| rs4913558 | 21 | 11058226 | G | C | 0.052 | 4.47×10^-10^ | 2.93×10^-8^ | BAGE2 | exonic |
| rs199979796 | 8 | 7679449 | G | C | 0.045 | 4.60×10^-10^ | 3.06×10^-8^ | DEFB105A | downstream |
| rs28462251 | 1 | 17086941 | C | T | 0.037 | 4.82×10^-10^ | 3.06×10^-8^ | MST1L | exonic |
| rs7188975 | 16 | 25137449 | A | T | 0.050 | 4.87×10^-10^ | 3.24×10^-8^ | LCMT1 | splicing |
| rs71614972 | 4 | 1389156 | T | C | 0.054 | 5.11×10^-10^ | 3.48×10^-8^ | CRIPAK | exonic |
| rs9824942 | 3 | 100432752 | A | T | 0.050 | 5.26×10^-10^ | 3.70×10^-8^ | TFG | intronic |
| rs144979264 | 16 | 1291597 | C | G | 0.059 | 5.27×10^-10^ | 3.70×10^-8^ | TPSAB1 | exonic |
| rs61625631 | 19 | 43865692 | G | A | 0.052 | 5.48×10^-10^ | 3.70×10^-8^ | CD177 | exonic |
| rs5742571 | 22 | 18842353 | G | A | 0.054 | 5.50×10^-10^ | 3.70×10^-8^ | LOC101929738,LOC101929537 | intergenic |
| rs201994930 | 9 | 45730218 | G | A | 0.045 | 5.55×10^-10^ | 3.77×10^-8^ | FAM27A,FAM27E2 | intergenic |
| rs8107149 | 19 | 37754086 | G | C | 0.054 | 5.72×10^-10^ | 3.85×10^-8^ | LOC101927667 | intronic |
| rs62539310 | 9 | 41954579 | G | A | 0.047 | 5.79×10^-10^ | 3.89×10^-8^ | MGC21881 | exonic |
| rs201470840 | 1 | 145301831 | A | G | 0.051 | 5.83×10^-10^ | 3.89×10^-8^ | NBPF10 | intronic |
| rs76197396 | 15 | 20645827 | A | G | 0.047 | 6.09×10^-10^ | 3.89×10^-8^ | HERC2P3 | exonic |
| rs12565078 | 1 | 145293515 | A | G | 0.053 | 6.15×10^-10^ | 4.14×10^-8^ | NBPF10 | exonic |
| rs4080365 | 9 | 33391479 | G | A | 0.055 | 6.56×10^-10^ | 4.16×10^-8^ | AQP7 | intronic |
| rs2495117 | 14 | 19585390 | A | G | 0.058 | 6.76×10^-10^ | 4.25×10^-8^ | POTEG | downstream |
| rs74220905 | 7 | 100550354 | C | A | 0.054 | 6.84×10^-10^ | 4.40×10^-8^ | MUC3A | exonic |
| rs28590552 | 16 | 33499035 | A | G | 0.049 | 7.41×10^-10^ | 4.46×10^-8^ | LOC101929031,LOC390714 | intergenic |
| rs28565257 | 16 | 33499100 | C | G | 0.049 | 7.41×10^-10^ | 4.81×10^-8^ | LOC101929031,LOC390714 | intergenic |
| rs11582087 | 1 | 152278856 | T | G | 0.042 | 7.91×10^-10^ | 4.89×10^-8^ | FLG | exonic |
| rs76859932 | 1 | 16973606 | G | T | 0.050 | 8.39×10^-10^ | 5.12×10^-8^ | MST1P2 | exonic |
| rs28371713 | 22 | 42524795 | A | G | 0.039 | 8.45×10^-10^ | 5.17×10^-8^ | CYP2D6 | exonic |
| rs775174499 | 7 | 100550348 | A | C | 0.052 | 8.53×10^-10^ | 5.22×10^-8^ | MUC3A | exonic |
| rs17157198 | 15 | 102516492 | C | G | 0.049 | 8.94×10^-10^ | 5.22×10^-8^ | WASH3P | exonic |
| rs2060872 | 15 | 20457244 | G | C | 0.041 | 8.95×10^-10^ | 5.22×10^-8^ | CHEK2P2 | intergenic |
| rs78450896 | 14 | 19585357 | A | G | 0.063 | 9.89×10^-10^ | 5.29×10^-8^ | POTEG | downstream |
| rs201725275 | 16 | 1278767 | C | T | 0.059 | 1.29×10^-9^ | 5.38×10^-8^ | TPSB2 | exonic |
| rs200978921 | 9 | 45730193 | T | G | 0.044 | 1.30×10^-9^ | 5.72×10^-8^ | FAM27A,  FAM27E2 | intergenic |
| rs201775100 | 1 | 248247363 | C | T | 0.046 | 1.34×10^-9^ | 6.00×10^-8^ | OR2L13 | intronic |
| rs61774959 | 1 | 1588717 | G | A | 0.056 | 1.34×10^-9^ | 6.86×10^-8^ | CDK11B | UTR5 |
| rs200533379 | 1 | 248247463 | C | T | 0.049 | 1.42×10^-9^ | 6.90×10^-8^ | OR2L13 | intronic |
| rs199836609 | 1 | 145301836 | A | G | 0.046 | 1.51×10^-9^ | 7.07×10^-8^ | NBPF10 | intronic |
| rs202107419 | 16 | 1279704 | A | G | 0.051 | 1.58×10^-9^ | 7.32×10^-8^ | TPSB2 | exonic |
| rs200630968 | 16 | 1279710 | G | A | 0.051 | 1.58×10^-9^ | 7.32×10^-8^ | TPSB2 | exonic |
| rs192643610 | 16 | 1279714 | A | G | 0.051 | 1.58×10^-9^ | 7.33×10^-8^ | TPSB2 | exonic |
| rs62581042 | 9 | 141069844 | T | C | 0.050 | 1.65×10^-9^ | 8.17×10^-8^ | TUBBP5 | exonic |
| rs76003715 | 19 | 50464023 | C | G | 0.035 | 1.67×10^-9^ | 8.56×10^-8^ | SIGLEC11 | exonic |
| rs61013791 | 8 | 12236292 | C | T | 0.053 | 1.73×10^-9^ | 8.59×10^-8^ | FAM66A | intronic |
| rs7520249 | 1 | 152192114 | C | T | 0.044 | 1.77×10^-9^ | 8.73×10^-8^ | HRNR | exonic |
| rs111474526 | 18 | 11644364 | G | A | 0.026 | 1.88×10^-9^ | 9.07×10^-8^ | SLC35G4,  GNAL | intergenic |
| rs179472 | 1 | 40230336 | C | G | 0.040 | 2.03×10^-9^ | 9.64×10^-8^ | BMP8B | exonic |
| rs78729591 | 11 | 56143795 | G | A | 0.096 | 2.17×10^-9^ | 9.81×10^-8^ | OR8U1 | exonic |
| rs202193035 | 11 | 56143803 | A | G | 0.096 | 2.17×10^-9^ | 9.81×10^-8^ | OR8U1 | exonic |
| rs200433243 | 11 | 56143804 | G | C | 0.096 | 2.17×10^-9^ | 9.81×10^-8^ | OR8U1 | exonic |
| rs201727202 | 11 | 56143807 | G | A | 0.096 | 2.17×10^-9^ | 9.97×10^-8^ | OR8U1 | exonic |
| rs367922647 | 3 | 195378126 | C | A | 0.045 | 2.62×10^-9^ | 1.55×10^-7^ | APOD,  SDHAP2 | intergenic |
| rs74969461 | 15 | 102515257 | C | T | 0.040 | 2.85×10^-9^ | 1.55×10^-7^ | WASH3P | exonic |
| rs61249862 | 21 | 10916290 | T | C | 0.045 | 2.93×10^-9^ | 1.55×10^-7^ | TPTE | intronic |
| rs149695852 | 19 | 54726833 | T | C | 0.054 | 3.09×10^-9^ | 1.55×10^-7^ | LILRB3 | exonic |
| rs141453251 | 19 | 54726839 | C | T | 0.054 | 3.09×10^-9^ | 1.60×10^-7^ | LILRB3 | exonic |
| rs138124247 | 19 | 54726843 | C | G | 0.054 | 3.09×10^-9^ | 1.61×10^-7^ | LILRB3 | exonic |
| rs114813697 | 19 | 54726861 | C | T | 0.054 | 3.09×10^-9^ | 1.63×10^-7^ | LILRB3 | UTR5 |
| rs61776760 | 1 | 1588737 | C | T | 0.055 | 3.13×10^-9^ | 1.88×10^-7^ | CDK11B | UTR5 |
| rs61776761 | 1 | 1588743 | A | T | 0.055 | 3.13×10^-9^ | 1.88×10^-7^ | CDK11B | UTR5 |
| rs151003 | 1 | 148903097 | C | A | 0.044 | 3.25×10^-9^ | 2.21×10^-7^ | LOC101927429,LOC645166 | intergenic |
| rs62038549 | 16 | 15083531 | C | A | 0.059 | 3.37×10^-9^ | 2.37×10^-7^ | PDXDC1 | intronic |
| rs61806689 | 1 | 121116678 | C | T | 0.052 | 3.47×10^-9^ | 2.51×10^-7^ | SRGAP2C | intronic |
| rs1318539 | 16 | 33710145 | T | A | 0.047 | 3.74×10^-9^ | 2.63×10^-7^ | LOC390714,SLC6A10PB | intergenic |
| rs80332440 | 19 | 54726816 | A | T | 0.054 | 3.76×10^-9^ | 2.64×10^-7^ | LILRB3 | exonic |
| rs532637 | 11 | 56468020 | T | C | 0.107 | 3.96×10^-9^ | 2.67×10^-7^ | OR9G1 | exonic |
| rs1052908 | 1 | 12855878 | A | C | 0.049 | 4.01×10^-9^ | 2.70×10^-7^ | PRAMEF1 | exonic |
| rs75333668 | 1 | 762320 | C | T | 0.023 | 4.13×10^-9^ | 2.75×10^-7^ | LINC00115 | exonic |
| rs1811313 | 16 | 33710177 | C | T | 0.049 | 4.22×10^-9^ | 2.78×10^-7^ | LOC390714,SLC6A10PB | intergenic |
| rs61995641 | 14 | 106805395 | G | A | 0.033 | 4.44×10^-9^ | 2.78×10^-7^ | IGHV4-31 | exonic |
| rs61995642 | 14 | 106805408 | C | T | 0.033 | 4.44×10^-9^ | 2.89×10^-7^ | IGHV4-31 | exonic |
| rs55679354 | 16 | 32077240 | T | C | 0.049 | 4.57×10^-9^ | 2.99×10^-7^ | LOC100289290,LOC100289574 | intergenic |
| rs766035137 | 16 | 32889182 | C | T | 0.049 | 4.57×10^-9^ | 3.02×10^-7^ | SLC6A10P | intronic |
| rs12602626 | 17 | 77089629 | T | A | 0.037 | 4.83×10^-9^ | 3.13×10^-7^ | RBFOX3 | intronic |
| rs12592066 | 15 | 20771825 | G | T | 0.051 | 5.37×10^-9^ | 3.17×10^-7^ | GOLGA8CP | intronic |
| rs74779225 | 22 | 17038752 | C | T | 0.050 | 5.55×10^-9^ | 3.18×10^-7^ | LOC101929674 | intronic |
| rs144574884 | 7 | 102246500 | A | G | 0.036 | 5.91×10^-9^ | 3.26×10^-7^ | RASA4 | intronic |
| rs77228815 | 15 | 84909385 | G | A | 0.050 | 6.31×10^-9^ | 3.88×10^-7^ | GOLGA6L4 | exonic |
| rs202043310 | 2 | 132044797 | C | A | 0.047 | 6.60×10^-9^ | 4.05×10^-7^ | LOC440910 | intronic |
| rs199598839 | 18 | 47492 | G | A | 0.039 | 6.97×10^-9^ | 4.28×10^-7^ | ROCK1P1 | intergenic |
| rs201493391 | 18 | 49034 | G | A | 0.039 | 6.97×10^-9^ | 4.39×10^-7^ | ROCK1P1 | intergenic |
| rs2095111 | 1 | 16975093 | C | T | 0.026 | 7.17×10^-9^ | 5.67×10^-7^ | MST1P2 | exonic |
| rs400522 | 16 | 28763776 | C | G | 0.034 | 8.97×10^-9^ | 6.14×10^-7^ | NPIPB9 | UTR5 |
| rs61814941 | 1 | 152188463 | G | A | 0.042 | 9.12×10^-9^ | 6.25×10^-7^ | HRNR | exonic |
| rs371602727 | 22 | 18775162 | C | T | 0.028 | 9.47×10^-9^ | 7.08×10^-7^ | GGT3P | intronic |
| rs57384288 | 11 | 1017773 | G | A | 0.048 | 9.65×10^-9^ | 7.98×10^-7^ | MUC6 | exonic |
| rs1529528 | 15 | 22345158 | T | C | 0.042 | 9.67×10^-9^ | 8.12×10^-7^ | LOC727924 | intronic |
| rs201982103 | 11 | 56143810 | T | C | 0.092 | 1.02×10^-8^ | 8.31×10^-7^ | OR8U1 | exonic |
| rs111512467 | 11 | 56143816 | G | C | 0.092 | 1.02×10^-8^ | 8.39×10^-7^ | OR8U1 | exonic |
| rs75484537 | 15 | 100340413 | C | T | 0.035 | 1.03×10^-8^ | 9.07×10^-7^ | DNM1P46 | exonic |
| rs141981140 | 1 | 148009349 | A | G | 0.043 | 1.04×10^-8^ | 9.36×10^-7^ | NBPF14 | exonic |
| rs201950480 | 3 | 195511686 | G | T | 0.026 | 1.12×10^-8^ | 9.36×10^-7^ | MUC4 | exonic |
| rs79852024 | 2 | 96610958 | G | T | 0.039 | 1.12×10^-8^ | 9.41×10^-7^ | ANKRD36C | intronic |
| rs73168325 | 7 | 100612786 | G | T | 0.097 | 1.16×10^-8^ | 1.02×10^-6^ | MUC12 | upstream |
| rs854250 | 16 | 32265022 | G | C | 0.049 | 1.32×10^-8^ | 1.05×10^-6^ | TP53TG3D | exonic |
| rs45473902 | 6 | 53784276 | A | T | 0.034 | 1.35×10^-8^ | 1.06×10^-6^ | LRRC1 | intronic |
| rs35978760 | 9 | 68438575 | C | G | 0.050 | 1.52×10^-8^ | 1.31×10^-6^ | LOC642236 | exonic |
| rs201024081 | 14 | 20181502 | C | T | 0.036 | 1.59×10^-8^ | 1.38×10^-6^ | OR11H2 | exonic |
| rs139993642 | 1 | 248637543 | C | T | 0.049 | 1.67×10^-8^ | 1.42×10^-6^ | OR2T3 | exonic |
| rs201798544 | 1 | 144193482 | G | A | 0.044 | 1.67×10^-8^ | 1.42×10^-6^ | NBPF8 | intronic |
| rs75720212 | 21 | 11059982 | A | G | 0.038 | 1.68×10^-8^ | 1.43×10^-6^ | BAGE2 | intronic |
| rs75799835 | 7 | 100552711 | T | A | 0.047 | 1.71E-08 | 1.49×10^-6^ | MUC3A | exonic |
| rs968322 | 1 | 47610293 | C | T | 0.048 | 1.80×10^-8^ | 1.51×10^-6^ | CYP4A22 | exonic |
| rs200169628 | 4 | 190989668 | C | G | 0.043 | 1.81×10^-8^ | 1.58×10^-6^ | DUX2 | exonic |
| rs76784555 | 15 | 79058445 | G | A | 0.031 | 1.93×10^-8^ | 1.77×10^-6^ | ADAMTS7 | exonic |
| rs2483047 | 1 | 16976032 | A | C | 0.027 | 1.95×10^-8^ | 1.82×10^-6^ | MST1P2 | intronic |
| rs13296 | 6 | 44218120 | G | A | 0.409 | 1.99×10^-8^ | 1.82×10^-6^ | HSP90AB1 | exonic |
| rs4116992 | 11 | 89510367 | T | G | 0.049 | 2.04×10^-8^ | 2.26×10^-6^ | TRIM77,TRIM49 | intergenic |
| rs76279546 | 22 | 17038766 | C | T | 0.049 | 2.12×10^-8^ | 2.27×10^-6^ | LOC101929674 | intronic |
| rs4333952 | 10 | 38939523 | T | C | 0.040 | 2.37×10^-8^ | 2.40×10^-6^ | LINC00999,ACTR3BP5 | intergenic |
| 24_13489409 | 24 | 13489409 | C | A | 0.031 | 2.37×10^-8^ | 2.45×10^-6^ | NA | intergenic |
| rs13000249 | 2 | 130951754 | G | T | 0.046 | 2.41×10^-8^ | 2.62×10^-6^ | TUBA3E | exonic |
| rs550939 | 16 | 21548095 | G | A | 0.039 | 2.66×10^-8^ | 3.12×10^-6^ | SLC7A5P2,LOC101927814 | intergenic |
| rs146937777 | 9 | 141069883 | G | A | 0.023 | 2.85×10^-8^ | 3.21×10^-6^ | TUBBP5 | exonic |
| rs141737044 | 14 | 24439209 | C | T | 0.033 | 3.10×10^-8^ | 3.37×10^-6^ | DHRS4L2 | UTR5 |
| rs61786893 | 1 | 143163731 | C | A | 0.037 | 3.27×10^-8^ | 3.45×10^-6^ | MIR3118-2 | upstream |
| rs45561438 | 10 | 16737167 | A | G | 0.032 | 3.42×10^-8^ | 3.67×10^-6^ | RSU1 | intronic |
| rs71638814 | 1 | 22313521 | G | T | 0.037 | 3.46×10^-8^ | 3.90×10^-6^ | CELA3B | intronic |
| rs28427082 | 10 | 29784004 | G | A | 0.045 | 3.59×10^-8^ | 3.97×10^-6^ | SVIL | intronic |
| rs782178558 | 5 | 140573844 | C | T | 0.036 | 3.64×10^-8^ | 4.66×10^-6^ | PCDHB10 | exonic |
| rs10418095 | 19 | 48692832 | C | A | 0.035 | 3.81×10^-8^ | 4.87×10^-6^ | C19orf68 | intronic |
| 16_83983866 | 16 | 83983866 | G | C | 0.044 | 3.82×10^-8^ | 5.08×10^-6^ | OSGIN1 | intronic |
| rs181730812 | 7 | 57187502 | A | G | 0.028 | 4.28×10^-8^ | 5.13×10^-6^ | ZNF479 | UTR3 |
| rs80216984 | 12 | 40899006 | C | A | 0.087 | 4.36×10^-8^ | 5.48×10^-6^ | MUC19 | intronic |
| rs1985842 | 22 | 42523409 | G | T | 0.034 | 4.38×10^-8^ | 5.84×10^-6^ | CYP2D6 | intronic |
| rs200002040 | 1 | 16916514 | C | T | 0.035 | 4.59×10^-8^ | 6.04×10^-6^ | NBPF1 | intronic |
| 24_13489514 | 24 | 13489514 | C | T | 0.022 | 4.64×10^-8^ | 6.04×10^-6^ | NA | intergenic |
| 23_118603844 | 23 | 118603844 | T | G | 0.044 | 4.65×10^-8^ | 6.21×10^-6^ | NA | intergenic |
| rs10148460 | 14 | 106791131 | A | G | 0.033 | 4.66×10^-8^ | 6.29×10^-6^ | IGHV3-30 | exonic |
| rs76070043 | 16 | 88798341 | T | C | 0.042 | 4.85×10^-8^ | 7.08×10^-6^ | LOC100289580 | intronic |
| rs113687113 | 16 | 33965655 | C | A | 0.034 | 4.95×10^-8^ | 7.28×10^-6^ | LINC00273,LOC100505948 | intergenic |
| rs138700801 | 1 | 12907496 | T | C | 0.029 | 5.26×10^-8^ | 7.29×10^-6^ | HNRNPCL1 | exonic |
| rs149187716 | 1 | 12855962 | C | T | 0.029 | 5.26×10^-8^ | 7.29×10^-6^ | PRAMEF1 | exonic |
| rs78163065 | 1 | 12907449 | C | G | 0.029 | 5.26×10^-8^ | 7.30×10^-6^ | HNRNPCL1 | exonic |
| rs41279490 | 1 | 12908080 | C | T | 0.029 | 5.26×10^-8^ | 7.36×10^-6^ | HNRNPCL1 | exonic |
| rs41279492 | 1 | 12908130 | C | T | 0.029 | 5.26×10^-8^ | 7.50×10^-6^ | HNRNPCL1 | exonic |
| rs12794886 | 11 | 62383715 | G | C | 0.101 | 5.32×10^-8^ | 7.95×10^-6^ | B3GAT3 | intronic |
| 16_88798334 | 16 | 88798334 | G | A | 0.036 | 6.72×10^-8^ | 7.95×10^-6^ | LOC100289580 | intronic |
| rs9510304 | 13 | 19447722 | C | G | 0.040 | 6.78×10^-8^ | 7.95×10^-6^ | ANKRD20A9P,RNU6-76P | intergenic |
| rs59808209 | 15 | 20467242 | C | T | 0.027 | 7.28×10^-8^ | 7.95×10^-6^ | NONE,CHEK2P2 | intergenic |
| rs201574565 | 2 | 97827830 | T | A | 0.039 | 7.31×10^-8^ | 7.95×10^-6^ | ANKRD36 | intronic |
| rs139914237 | 18 | 11644581 | G | A | 0.020 | 7.45×10^-8^ | 8.04×10^-6^ | SLC35G4,GNAL | intergenic |
| rs10794633 | 15 | 20465182 | T | A | 0.031 | 7.59×10^-8^ | 8.04×10^-6^ | CHEK2P2 | intergenic |
| rs55638883 | 1 | 22329220 | G | A | 0.031 | 8.28×10^-8^ | 8.28×10^-6^ | CELA3A | intronic |
| 17_36365172 | 17 | 36365172 | C | T | 0.034 | 8.31×10^-8^ | 8.66×10^-6^ | LOC440434 | exonic |
| rs2571124 | 19 | 44898662 | C | T | 0.036 | 8.41×10^-8^ | 8.77×10^-6^ | ZNF285 | intronic |
| rs4570422 | 1 | 16974869 | G | C | 0.024 | 8.48×10^-8^ | 8.77×10^-6^ | MST1P2 | exonic |
| rs1295463 | 1 | 47582246 | C | T | 0.039 | 8.49×10^-8^ | 8.77×10^-6^ | CYP4Z1 | intronic |
| rs10794632 | 15 | 20465167 | A | T | 0.030 | 9.76×10^-8^ | 8.93×10^-6^ | CHEK2P2 | intergenic |
| rs35159134 | 13 | 24471048 | C | T | 0.049 | 9.84×10^-8^ | 9.18×10^-6^ | C1QTNF9B | exonic |
| rs781981016 | 4 | 190989533 | G | A | 0.019 | 1.02×10^-7^ | 9.70×10^-6^ | DUX2 | exonic |
| 4_190989539 | 4 | 190989539 | G | T | 0.019 | 1.02×10^-7^ | 1.00×10^-5^ | DUX2 | exonic |
| rs71246352 | 1 | 144855782 | C | T | 0.031 | 1.02×10^-7^ | 1.01×10^-5^ | PDE4DIP | exonic |
| rs74584169 | 11 | 89774236 | C | G | 0.049 | 1.03×10^-7^ | 1.02×10^-5^ | TRIM49C | exonic |
| rs75182069 | 7 | 66764392 | T | C | 0.038 | 1.16×10^-7^ | 1.04×10^-5^ | PMS2P4 | exonic |
| rs200427293 | 1 | 248737293 | G | A | 0.042 | 1.16×10^-7^ | 1.06×10^-5^ | OR2T34 | exonic |
| rs201475070 | 1 | 248737294 | C | G | 0.042 | 1.16×10^-7^ | 1.06×10^-5^ | OR2T34 | exonic |
| rs1769774 | 1 | 12854428 | C | T | 0.044 | 1.29×10^-7^ | 1.09×10^-5^ | PRAMEF1 | exonic |
| rs41288999 | 10 | 30915906 | A | G | 0.016 | 1.37×10^-7^ | 1.11×10^-5^ | LYZL2 | intronic |
| rs6931014 | 6 | 58246143 | A | G | 0.494 | 1.38×10^-7^ | 1.16×10^-5^ | GUSBP4 | downstream |
| rs71642766 | 1 | 222649491 | C | G | 0.037 | 1.38×10^-7^ | 1.18×10^-5^ | TRNAT19,LOC101926901 | intergenic |
| rs1182136 | 7 | 2966445 | T | G | 0.024 | 1.46×10^-7^ | 1.20×10^-5^ | CARD11 | intronic |
| rs13329439 | 15 | 71457127 | T | A | 0.044 | 1.50×10^-7^ | 1.20×10^-5^ | THSD4 | intronic |
| rs4000678 | 1 | 16894323 | C | A | 0.027 | 1.51×10^-7^ | 1.27×10^-5^ | NBPF1 | intronic |
| rs75766753 | 7 | 76669333 | A | C | 0.036 | 1.57×10^-7^ | 1.29×10^-5^ | LOC100132832 | exonic |
| rs3970662 | 22 | 18863465 | G | A | 0.043 | 1.59×10^-7^ | 1.54×10^-5^ | LOC100996432 | intronic |
| rs201332355 | 9 | 135895382 | T | C | 0.043 | 1.70×10^-7^ | 1.56×10^-5^ | GFI1B,GTF3C5 | intergenic |
| rs148138544 | 10 | 81373674 | T | C | 0.025 | 1.77×10^-7^ | 1.65×10^-5^ | SFTPA1 | exonic |
| rs16881817 | 7 | 22532260 | A | G | 0.044 | 1.79×10^-7^ | 1.69×10^-5^ | STEAP1B | exonic |
| rs201849753 | 1 | 741267 | T | C | 0.025 | 1.86×10^-7^ | 1.70×10^-5^ | FAM87B | intergenic |
| rs113727842 | 6 | 161032668 | A | G | 0.049 | 1.94×10^-7^ | 1.85×10^-5^ | LPA | exonic |
| rs61786895 | 1 | 143163799 | G | A | 0.037 | 2.01×10^-7^ | 1.89×10^-5^ | MIR3118-2 | exonic |
| rs113880309 | 21 | 14410672 | G | C | 0.025 | 2.07×10^-7^ | 1.98×10^-5^ | ANKRD30BP2 | exonic |
| rs201138873 | 1 | 121115981 | C | T | 0.039 | 2.27×10^-7^ | 1.99×10^-5^ | SRGAP2C | exonic |
| rs11202256 | 10 | 81993489 | C | T | 0.046 | 2.32×10^-7^ | 2.16×10^-5^ | LINC00857,LOC100130698 | intergenic |
| rs3778731 | 7 | 154587507 | G | A | 0.042 | 2.66×10^-7^ | 2.25×10^-5^ | DPP6 | intronic |
| rs13236282 | 7 | 5922056 | T | C | 0.462 | 2.67×10^-7^ | 2.27×10^-5^ | OCM | intronic |
| rs9880320 | 3 | 190594309 | G | A | 0.039 | 2.71×10^-7^ | 2.28×10^-5^ | GMNC | intronic |
| rs13330343 | 16 | 32489447 | G | A | 0.041 | 2.82×10^-7^ | 2.31×10^-5^ | TP53TG3 | intergenic |
| rs200422093 | 19 | 56273285 | A | G | 0.033 | 2.88×10^-7^ | 2.42×10^-5^ | RFPL4A | exonic |
| rs609553 | 16 | 28354090 | C | G | 0.045 | 2.95×10^-7^ | 2.51×10^-5^ | NPIPB6 | exonic |
| rs77178122 | 20 | 29633854 | G | A | 0.039 | 2.98×10^-7^ | 2.55×10^-5^ | FRG1B | intronic |
| rs4925787 | 1 | 248112627 | T | C | 0.047 | 3.02×10^-7^ | 2.55×10^-5^ | OR2L8 | exonic |
| 24_13489448 | 24 | 13489448 | A | T | 0.024 | 3.06×10^-7^ | 2.60×10^-5^ | NA | intergenic |
| rs11160825 | 14 | 105410411 | C | T | 0.044 | 3.17×10^-7^ | 2.61×10^-5^ | AHNAK2 | exonic |
| rs13330639 | 16 | 32489408 | C | T | 0.040 | 3.22×10^-7^ | 2.69×10^-5^ | TP53TG3 | intergenic |
| rs4070337 | 10 | 38938479 | C | A | 0.059 | 3.41×10^-7^ | 2.74×10^-5^ | LINC00999,ACTR3BP5 | intergenic |
| rs11486026 | 1 | 1635193 | T | C | 0.023 | 3.47×10^-7^ | 2.74×10^-5^ | CDK11A | intronic |
| rs200982240 | 1 | 1638925 | C | T | 0.023 | 3.47×10^-7^ | 2.74×10^-5^ | CDK11A | exonic |
| rs9764179 | 5 | 21483636 | G | C | 0.039 | 3.57×10^-7^ | 2.82×10^-5^ | LOC100506217 | exonic |
| rs78773460 | 10 | 29783908 | A | G | 0.041 | 3.61×10^-7^ | 2.92×10^-5^ | SVIL | exonic |
| rs62472905 | 7 | 62708659 | G | A | 0.041 | 3.73×10^-7^ | 2.95×10^-5^ | ZNF733P | intergenic |
| rs766350584 | 7 | 100550318 | G | A | 0.044 | 3.86×10^-7^ | 2.99×10^-5^ | MUC3A | exonic |
| rs747914533 | 7 | 100550312 | T | C | 0.043 | 3.94×10^-7^ | 3.09×10^-5^ | MUC3A | exonic |
| rs2593292 | 15 | 76078022 | T | G | 0.019 | 4.05×10^-7^ | 3.09×10^-5^ | LOC441728 | intronic |
| rs73055190 | 19 | 53818564 | C | T | 0.030 | 4.08×10^-7^ | 3.17×10^-5^ | BIRC8,ZNF845 | intergenic |
| rs10439122 | 19 | 53818603 | G | A | 0.030 | 4.08×10^-7^ | 3.19×10^-5^ | BIRC8,ZNF845 | intergenic |
| rs9694631 | 8 | 12236652 | C | G | 0.032 | 4.17×10^-7^ | 3.27×10^-5^ | FAM66A | nintronic |
| rs79090643 | 1 | 143163708 | C | T | 0.031 | 4.22×10^-7^ | 3.29×10^-5^ | MIR3118-2 | upstream |
| rs7712666 | 5 | 177548085 | C | A | 0.044 | 4.40×10^-7^ | 3.32×10^-5^ | N4BP3 | intronic |
| rs140005209 | 10 | 30915897 | A | G | 0.015 | 4.46×10^-7^ | 3.32×10^-5^ | LYZL2 | intronic |
| rs2550233 | 3 | 195453064 | G | T | 0.349 | 4.62×10^-7^ | 3.38×10^-5^ | MUC20 | exonic |
| rs7246746 | 19 | 54743758 | C | A | 0.039 | 4.72×10^-7^ | 3.42×10^-5^ | LILRA6 | intronic |
| rs58725160 | 1 | 1986737 | A | G | 0.020 | 4.78×10^-7^ | 3.51×10^-5^ | PRKCZ | intronic |

|  |
| --- |

Chr: chromosome, MAF: minor allele frequency

*HNRNPCL1* rs848418, *LOC100132147* rs915324, *LOC100132147* rs1759228, *MST1P2* rs1360575, *LOC101929455* rs55646423, *PDE4DIP* rs3863691 and rs2590121, *NOTCH2NL* rs8002, *NBPF10* rs5020524, *NBPF14* rs11485976, *LINC00869* rs587319, *SRGAP2* rs201036189, *FRG1* rs78653319, *LOC100996481* rs518931, *LOC100996481* rs562762, *MUC3A* rs74588241, rs77667788, rs76951301, rs73714276,and rs75592954, *MUC12* rs75162633, *PRSS1* rs201825825, *TRBC2* rs782096363 and rs782462756, *KMT2C* rs2479172, *CA2,REXO1L1* rs371583881 and rs374417807, *LOC101928301* rs113087405, rs79475537,and rs796130919, *LOC644249* rs112952566, rs1906354 an rs77458778, *LOC642236* rs4568707, *ACTR3BP5* rs4436477 and rs4474366, *MUC6* rs34740154, rs35469308, rs113508205, rs113559934, rs74632841, rs34375705, rs34053383, rs199579978, rs35621365, rs77885750, rs34649796, rs74202058, rs61869004, rs34844844, rs33943903, rs55903826, rs76686156, rs78943453, rs34912894, rs34095361, rs77359624, rs75637734, rs78819924, rs61869009 and rs61869010, *SAA2* rs1136766, *OR4C3* rs77470587, rs72911452, rs75900655, rs75647397, rs73463988, *OR9G1* rs1704284, *LOC100288778* rs71260408

*LOC100505679* rs13329312, *LINC00273* rs375920374, rs199951204, rs763760731, rs72799208 and rs796492701, *MAP2K3* rs72840055, rs72840057, rs62057721, *KCNJ12* rs1657745, *FAM27L* rs9285743, *ZNF676* rs80146743,

*LOC101926935* rs79754881, rs11699441 and rs6037279, *FRG1B* rs10439604, rs9647043 and rs4006816, *MLLT10P1* rs62206782, *LOC101926954* rs610070, rs3915340, 24_13489425, 24_13489507, 24_13489508, 24_13489515, *MUC6* rs747429892, *OR4C3* rs75498992, rs79042268, *NBPF1* rs9728933, *MUC3A* rs74588241 have strong linkage disequilibrium.

**Table S10** Single nucleotide polymorphism correlated to platelet count

| SNP | Chr | Position | Ref Allele | Min Allele | MAF | Bonferroni *p* value | FDR  *p* value | Gene | Position |
| --- | --- | --- | --- | --- | --- | --- | --- | --- | --- |
| rs41274498 | 1 | 11126697 | G | C | 0.051 | 2.16×10^-6^ | 0.168 | EXOSC10 | UTR3 |
| rs141784567 | 3 | 53263112 | T | C | 0.014 | 4.13×10^-6^ | 0.168 | TKT | exonic |
| rs10486527 | 7 | 33380673 | T | C | 0.106 | 4.71×10^-6^ | 0.168 | BBS9 | intronic |
| rs567845071 | 15 | 29034622 | A | G | 0.011 | 8.58×10^-6^ | 0.208 | LOC646278;LOC100289656 | upstream; |
| rs11981364 | 7 | 33380598 | G | T | 0.109 | 9.70×10^-6^ | 0.208 | BBS9 | intronic |
| rs17116471 | 10 | 105840422 | T | C | 0.077 | 2.26×10^-5^ | 0.374 | COL17A1 | exonic |
| rs3750319 | 9 | 131670919 | C | T | 0.311 | 2.54×10^-5^ | 0.374 | LRRC8A | exonic |
| rs59649940 | 2 | 86400791 | T | C | 0.100 | 2.80×10^-5^ | 0.374 | IMMT | exonic |
| rs13101652 | 4 | 9707682 | G | A | 0.149 | 3.61×10^-5^ | 0.378 | LOC101928948,DRD5 | intergenic |
| rs2148911 | 1 | 20395587 | G | A | 0.251 | 4.82×10^-5^ | 0.378 | PLA2G5 | intronic |
| rs78070589 | 1 | 53932181 | A | G | 0.169 | 5.04×10^-5^ | 0.378 | DMRTB1 | intronic |
| rs61254540 | 2 | 86272309 | G | A | 0.094 | 5.45×10^-5^ | 0.378 | POLR1A | intronic |
| rs61185790 | 2 | 86272350 | C | G | 0.094 | 5.45×10^-5^ | 0.378 | POLR1A | intronic |
| rs10184159 | 2 | 86276395 | C | G | 0.094 | 5.45×10^-5^ | 0.378 | POLR1A | intronic |
| rs2288116 | 2 | 86302409 | A | G | 0.094 | 5.45×10^-5^ | 0.378 | POLR1A | intronic |
| rs2280270 | 17 | 75205562 | C | T | 0.089 | 6.47×10^-5^ | 0.378 | SEC14L1 | intronic |
| rs2304840 | 17 | 78085911 | G | A | 0.043 | 6.49×10^-5^ | 0.378 | GAA | intronic |
| rs11190326 | 10 | 101690415 | G | A | 0.017 | 7.13×10^-5^ | 0.378 | LOC101927371 | exonic |
| rs1286823 | 1 | 62257036 | A | C | 0.026 | 7.76×10^-5^ | 0.378 | INADL | exonic |
| rs2987791 | 10 | 135370060 | C | G | 0.166 | 8.28×10^-5^ | 0.378 | SYCE1 | intronic |
| rs3020502 | 10 | 135381623 | C | T | 0.166 | 8.28×10^-5^ | 0.378 | SPRNP1 | exonic |
| rs3020503 | 10 | 135381766 | T | C | 0.166 | 8.28×10^-5^ | 0.378 | SPRNP1 | exonic |
| rs10141024 | 14 | 94391699 | A | G | 0.394 | 8.44×10^-5^ | 0.378 | FAM181A | exonic |
| rs183698747 | 8 | 25237761 | G | C | 0.011 | 8.52×10^-5^ | 0.378 | DOCK5 | intronic |
| rs2515641 | 10 | 135351362 | C | T | 0.166 | 8.83×10^-5^ | 0.378 | CYP2E1 | exonic |
| rs982146 | 4 | 83822334 | G | A | 0.497 | 9.34×10^-5^ | 0.384 | THAP9 | intronic |

Chr: chromosome, Ref allele: reference allele, Min: minor allele, MAF: minor allele frequency, FDR: false discovery rate
